# Supplementary material for: Comparative transcriptome analysis during seeds development between two soybean cultivars
Source: PeerJ. 2021 Mar 1;9:e10772. doi: 10.7717/peerj.10772 (PMC7931715; doi:10.7717/peerj.10772)

Figure S1 The saturation curves of all of the samples in A7 and A35 (1).

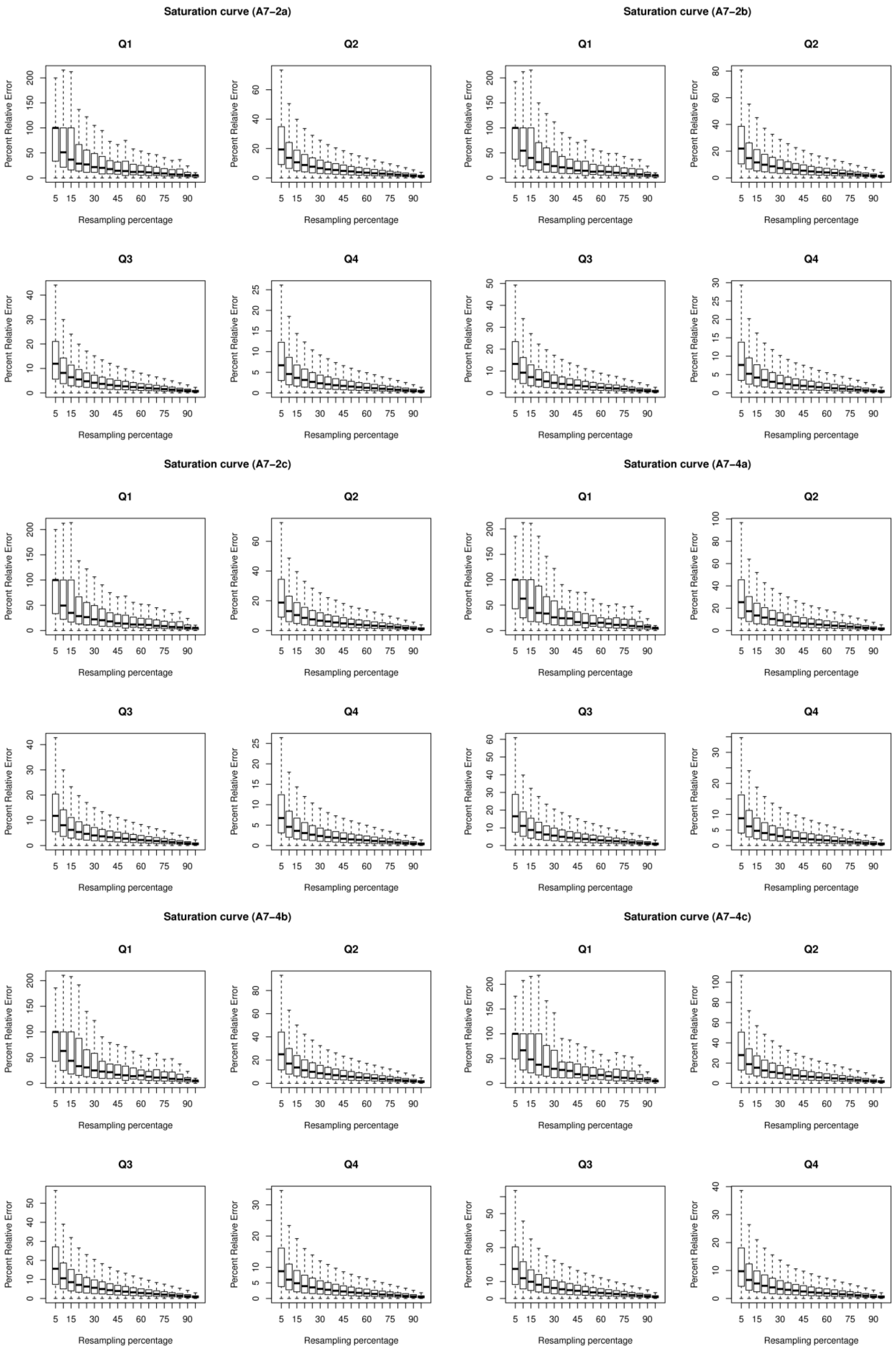

Figure S1. The saturation curves of all of the samples in A7 and A35 (2).

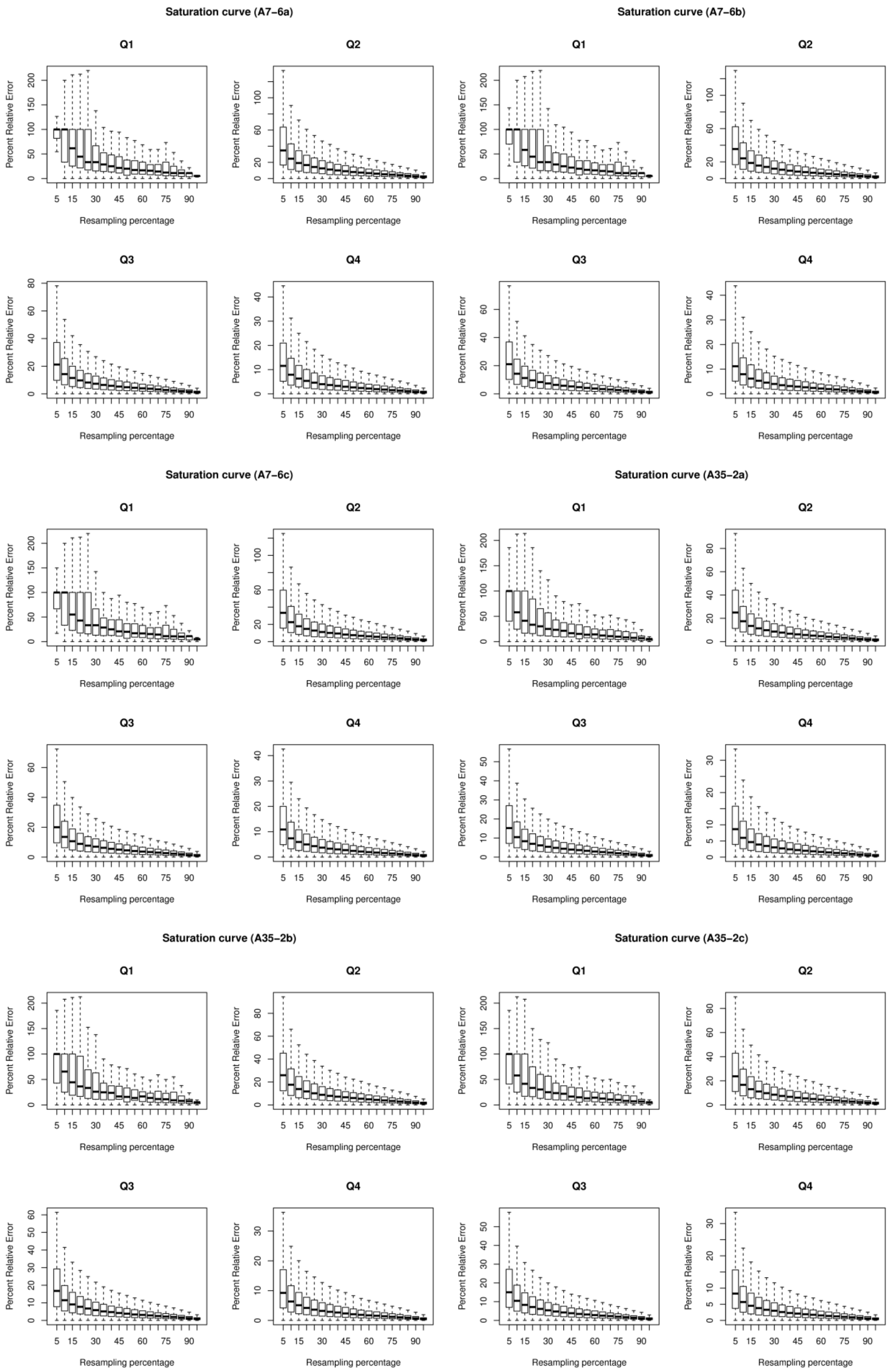

Figure S1. The saturation curves of all of the samples in A7 and A35 (3).

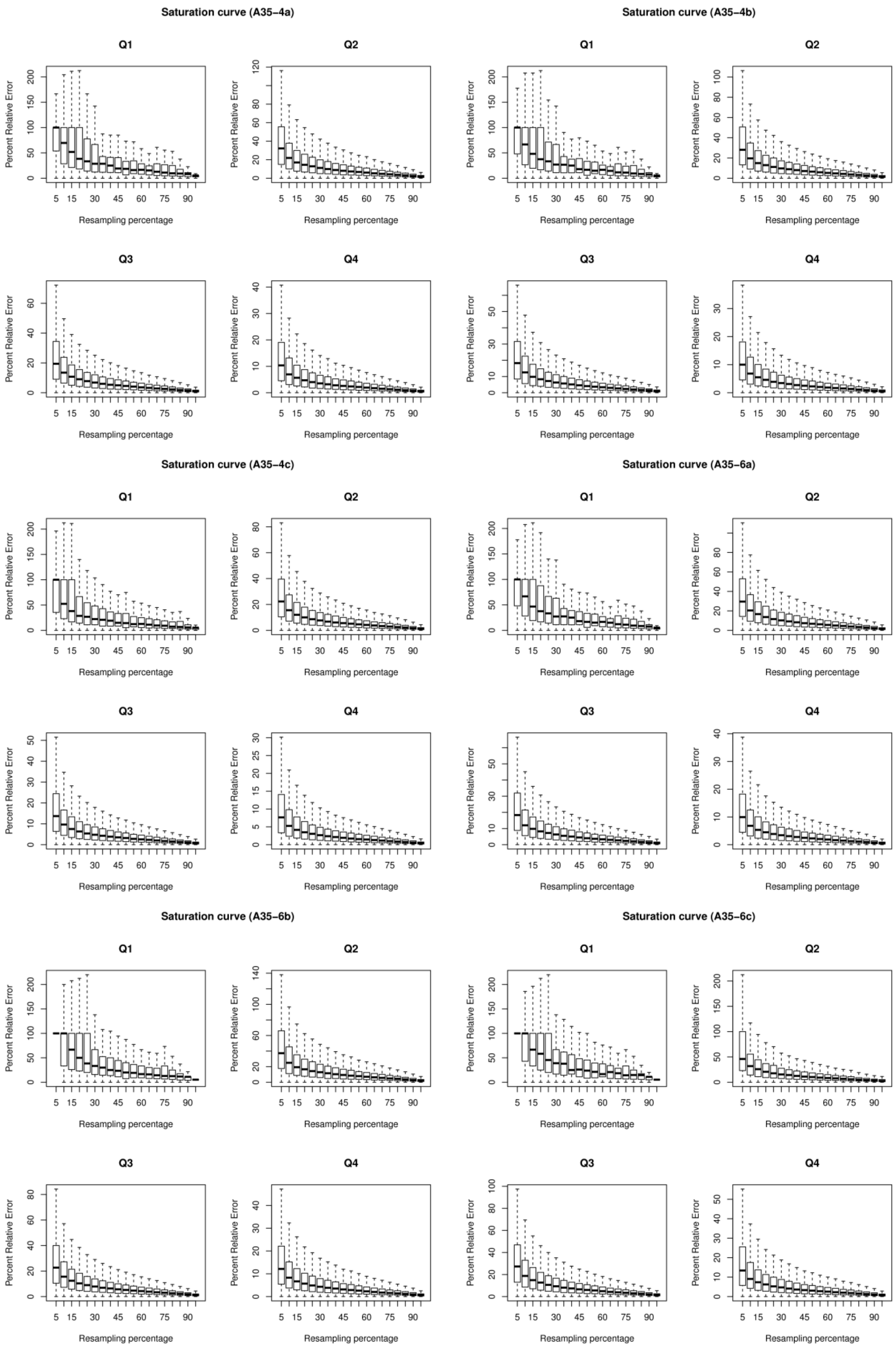

Figure S2 The evenness curves of samples in A7 and A35 (1).

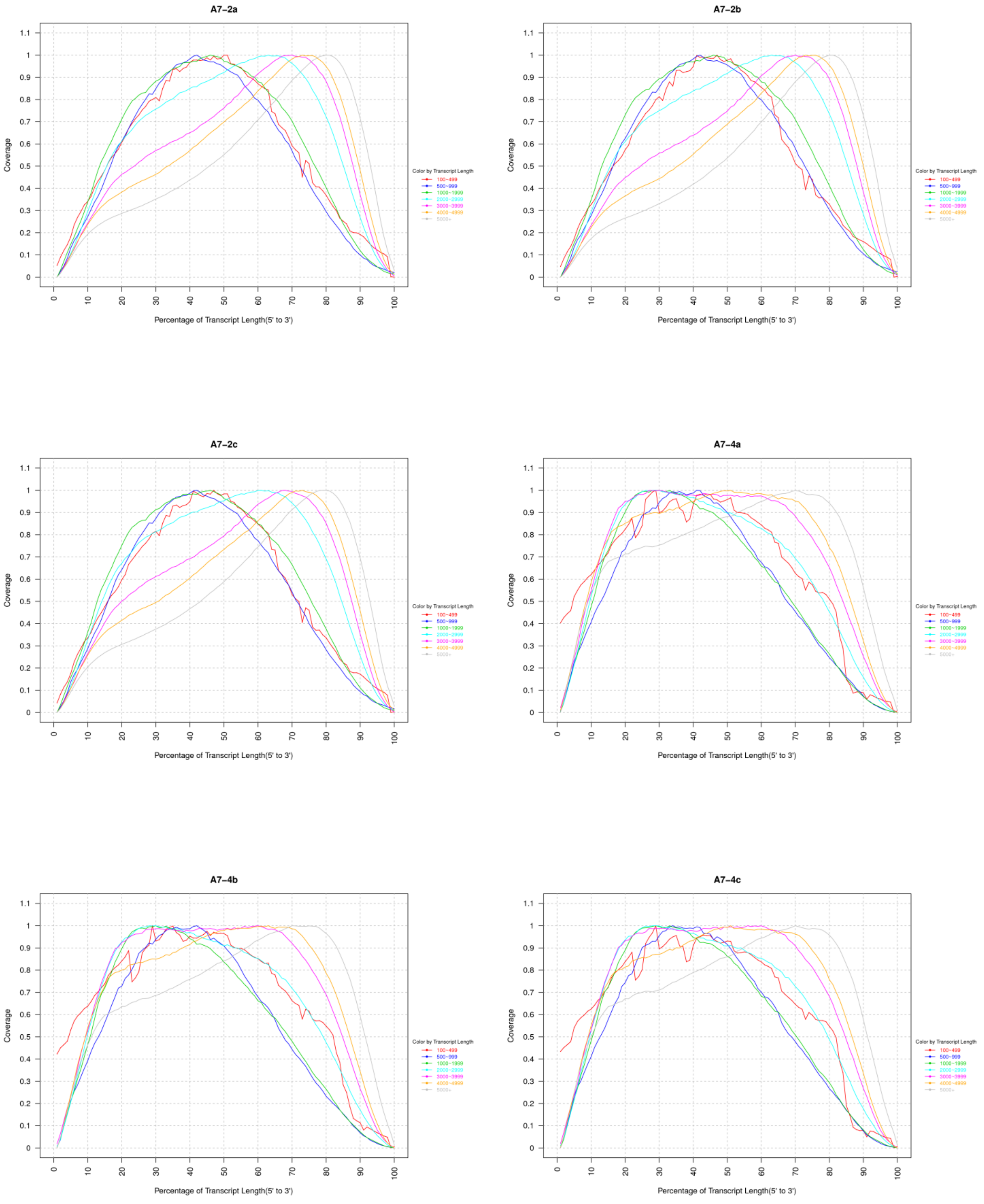

Figure S2 The evenness curves of samples in A7 and A35 (2).

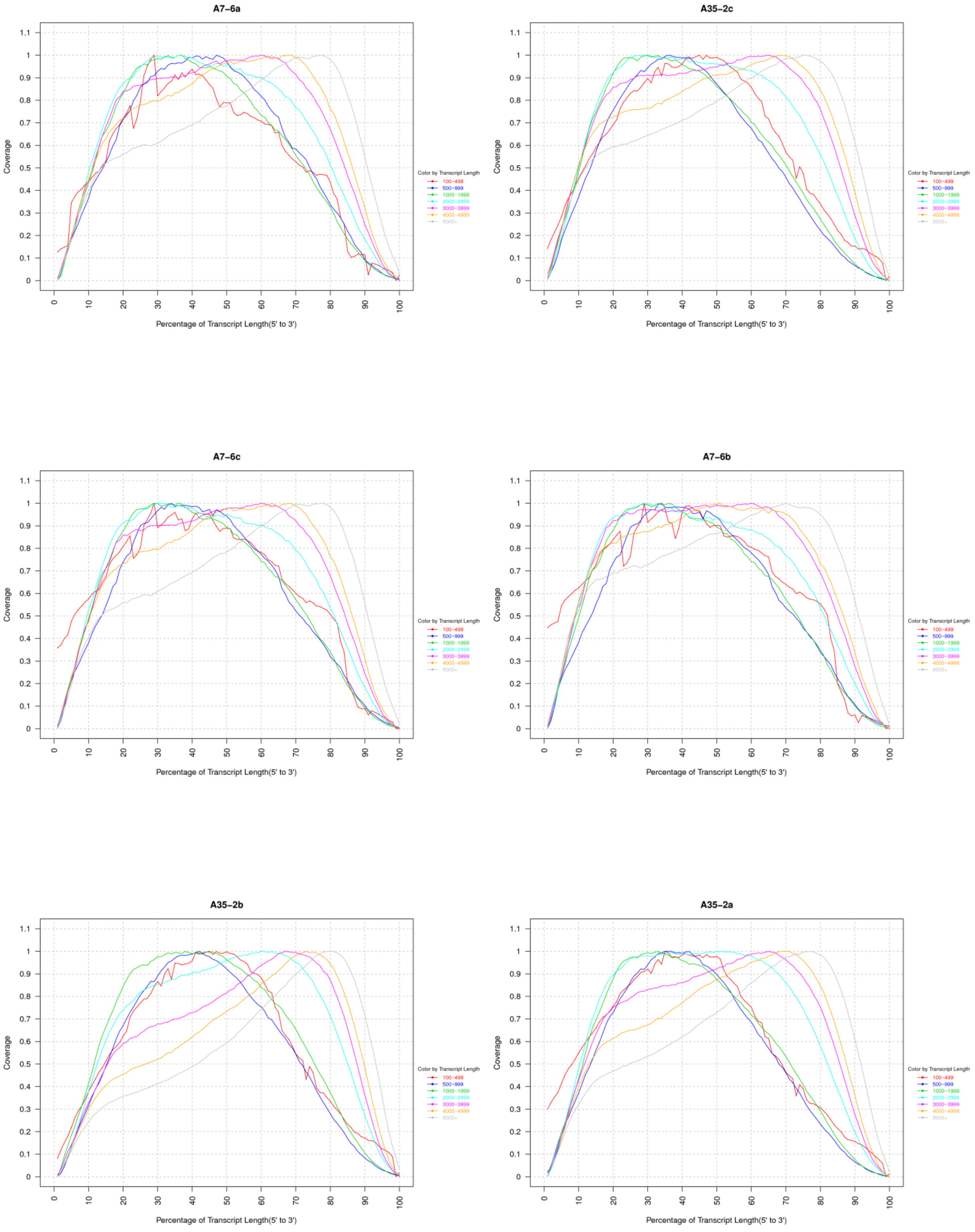

Figure S2 The evenness curves of samples in A7 and A35 (3)

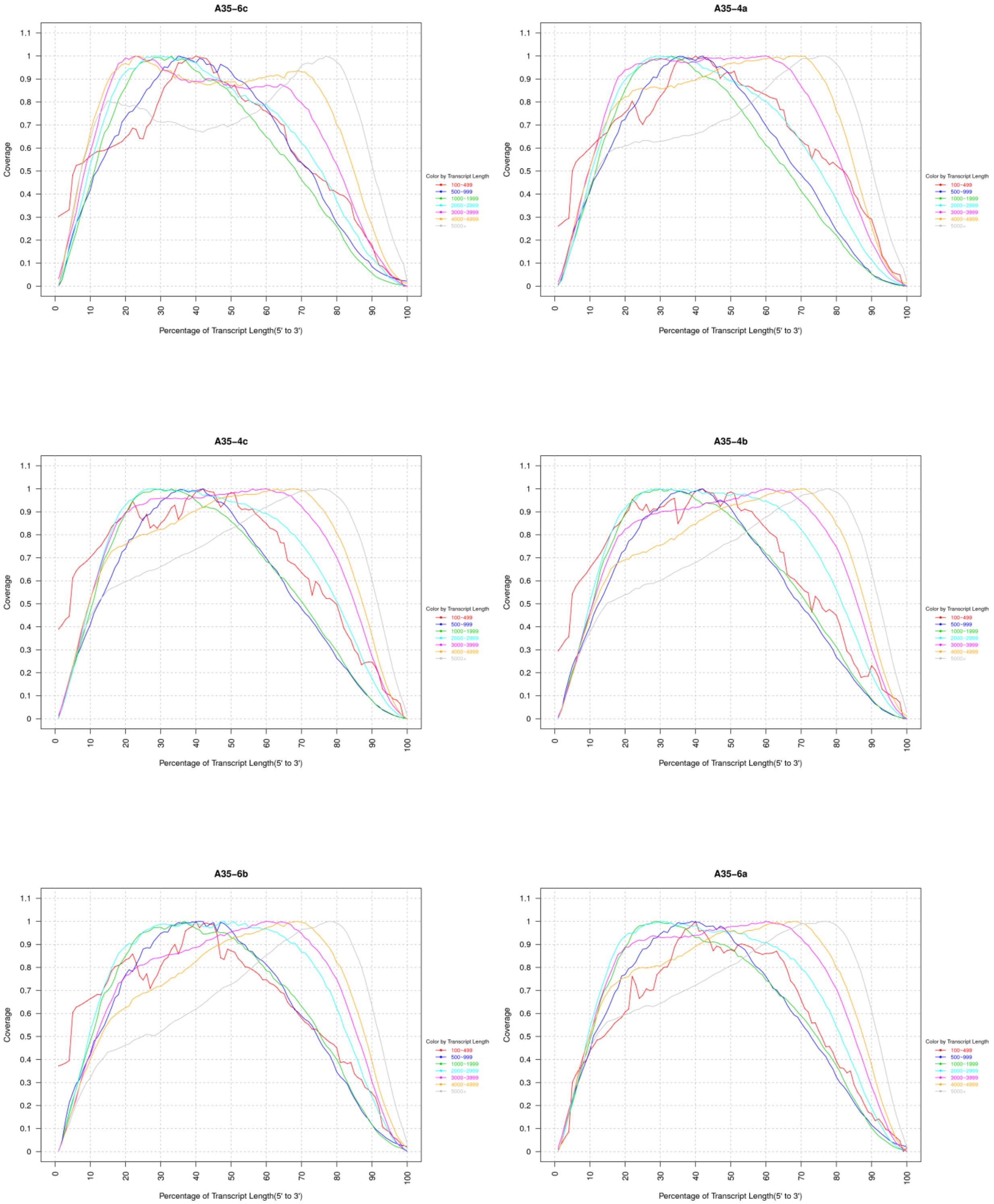

Figure S3 The correlation analysis of samples in A7 and A35 (1).

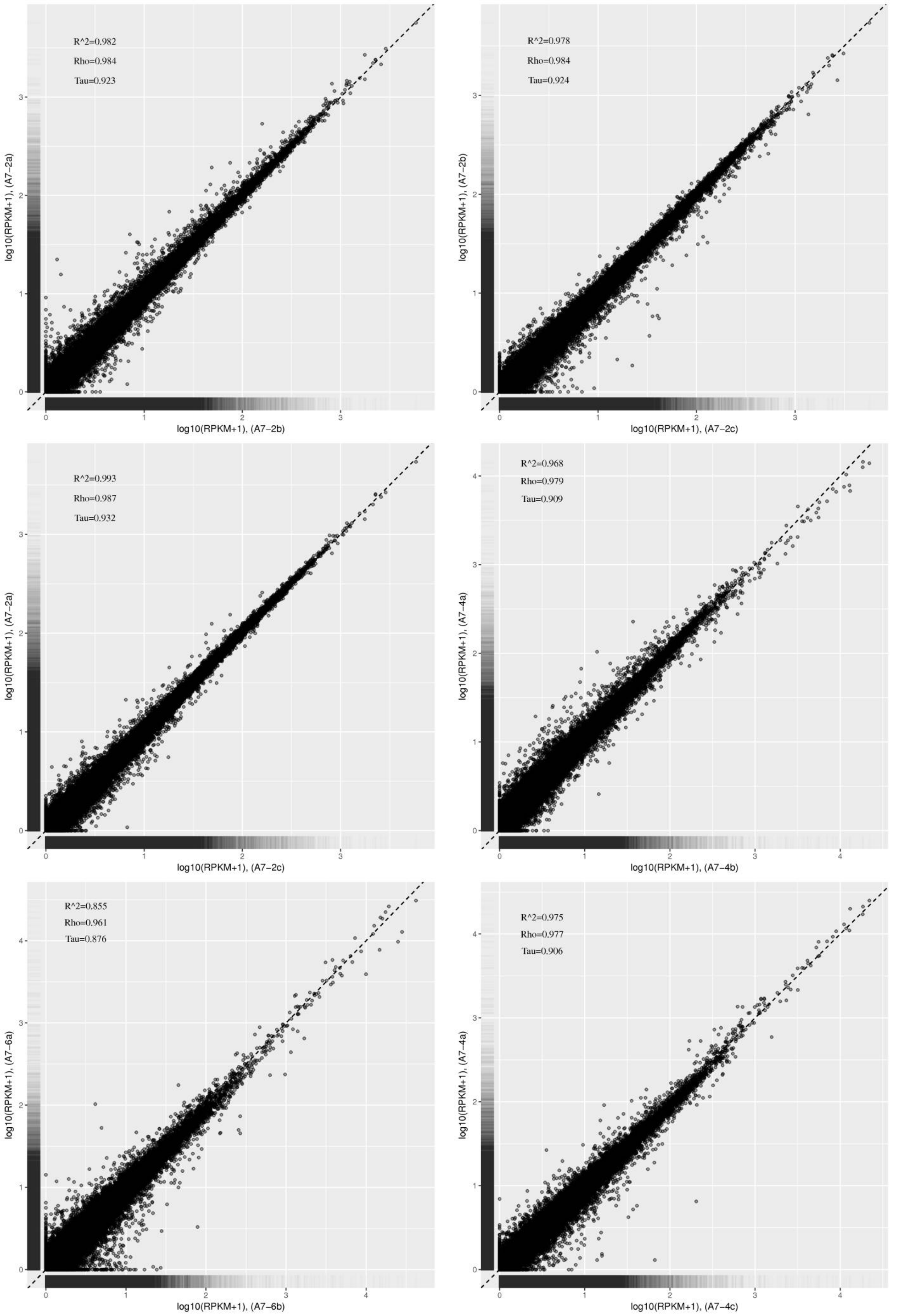

Figure S3. The correlation analysis of samples in A7 and A35 (2)

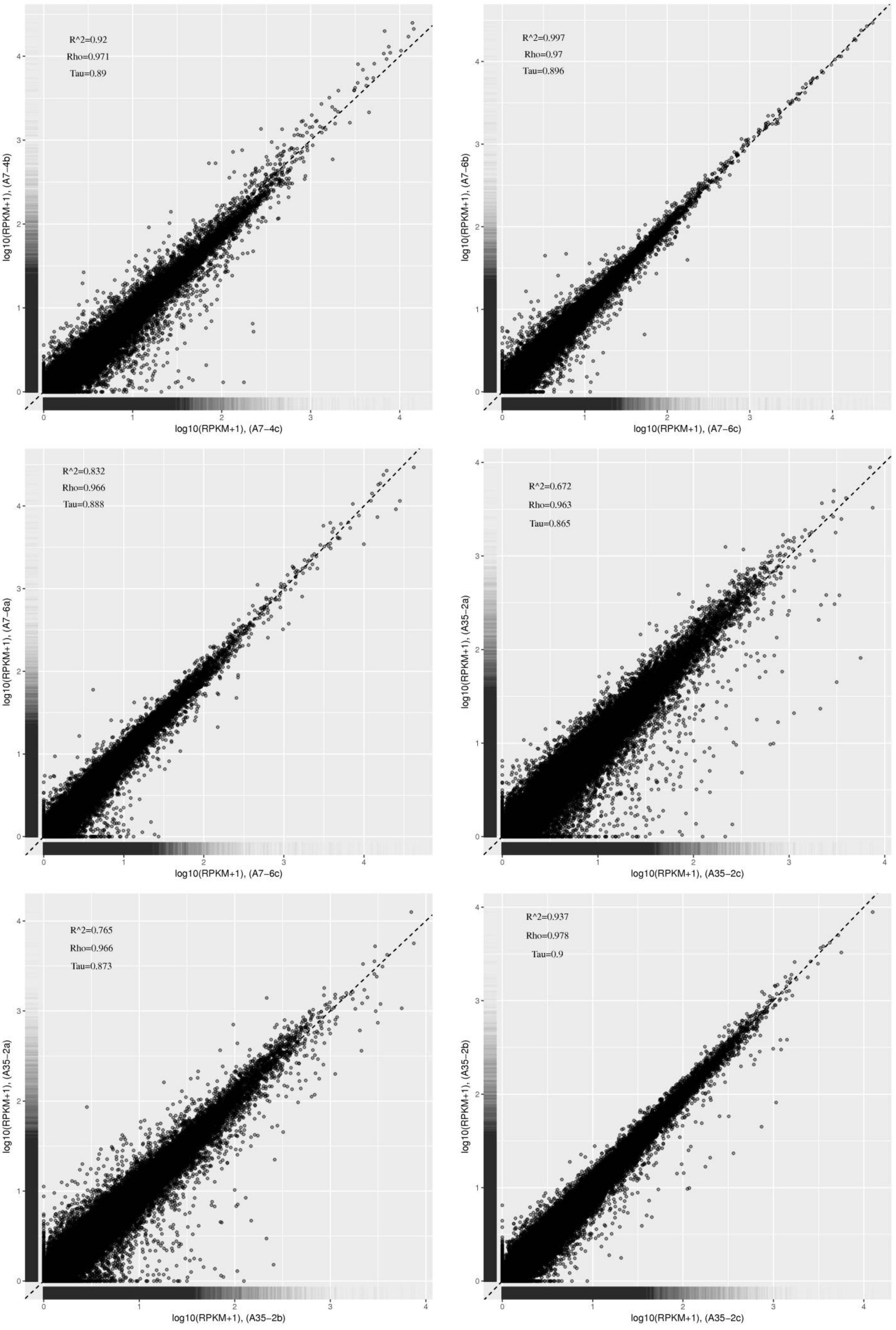

Figure S3. The correlation analysis of samples in A7 and A35 (3).

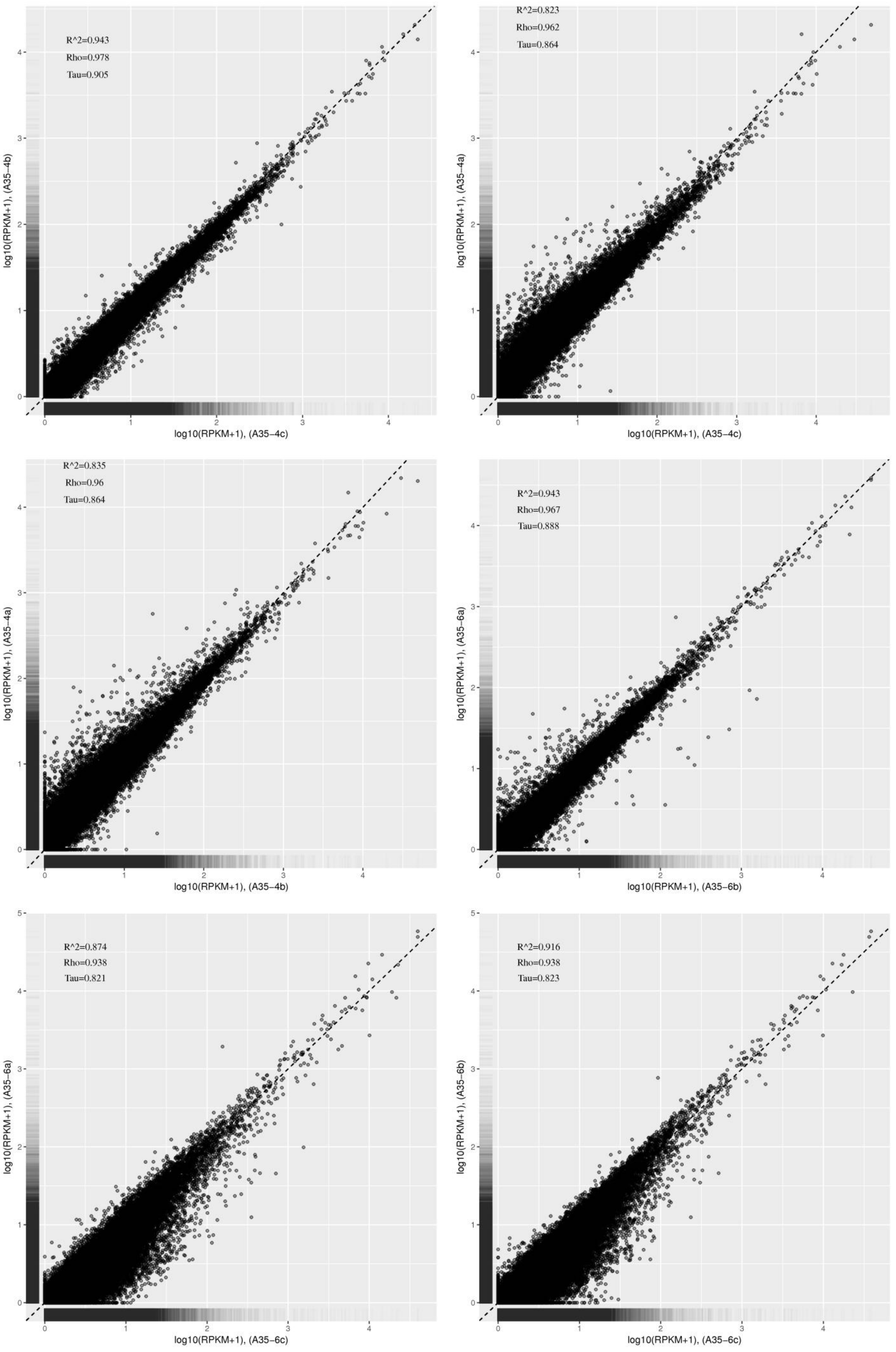

Figure S4. Volcano plots of DEGs. The up-regulated and down-regulated DEGs were shown in red and blue, respectively. The x-axis represented the fold change of DEGs in different samples. The y-axis represented the statistical significance of gene expression differences.

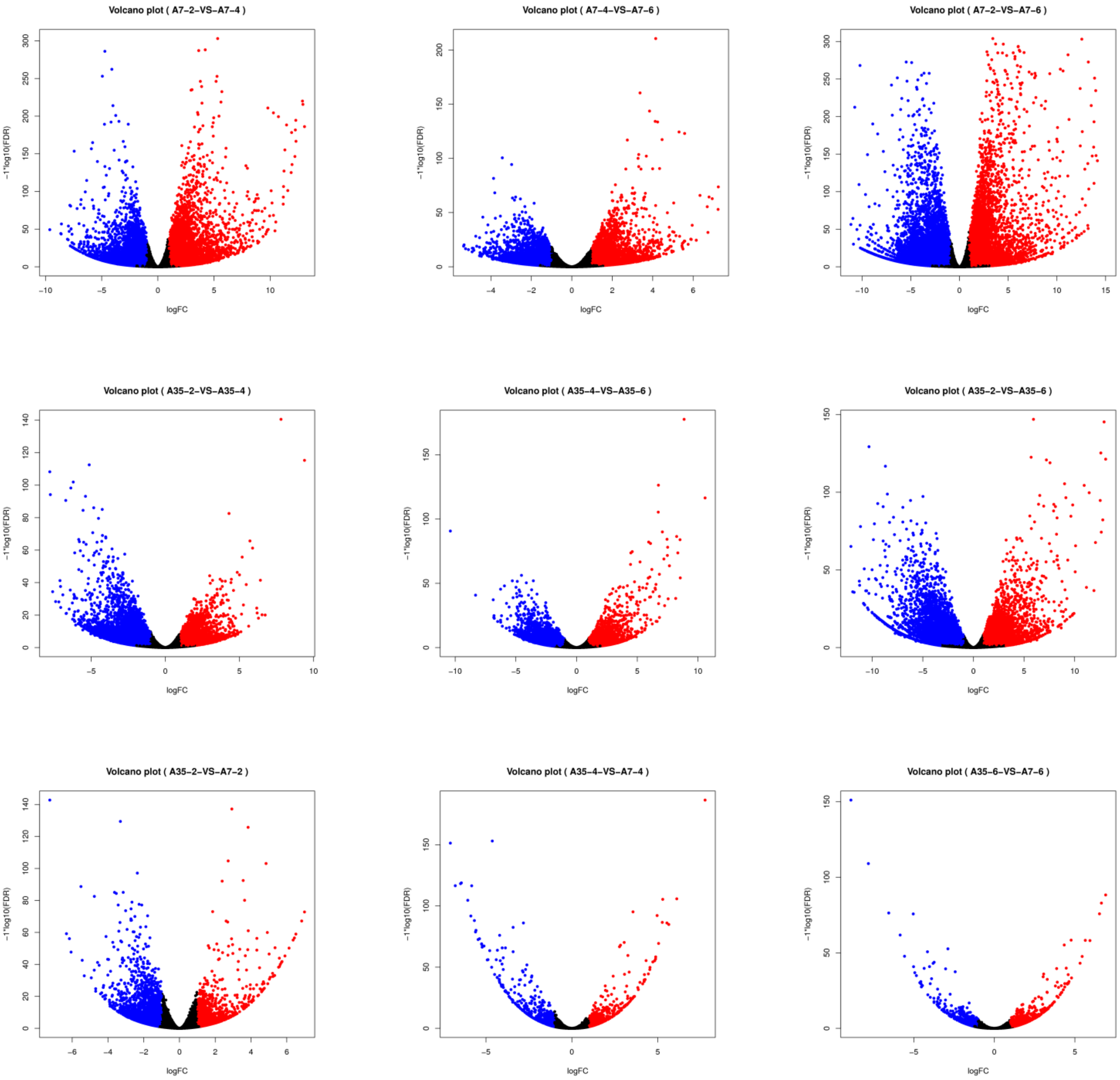

Figure S5. The principal component analysis (PCA) analysis of samples in A7 and A35.

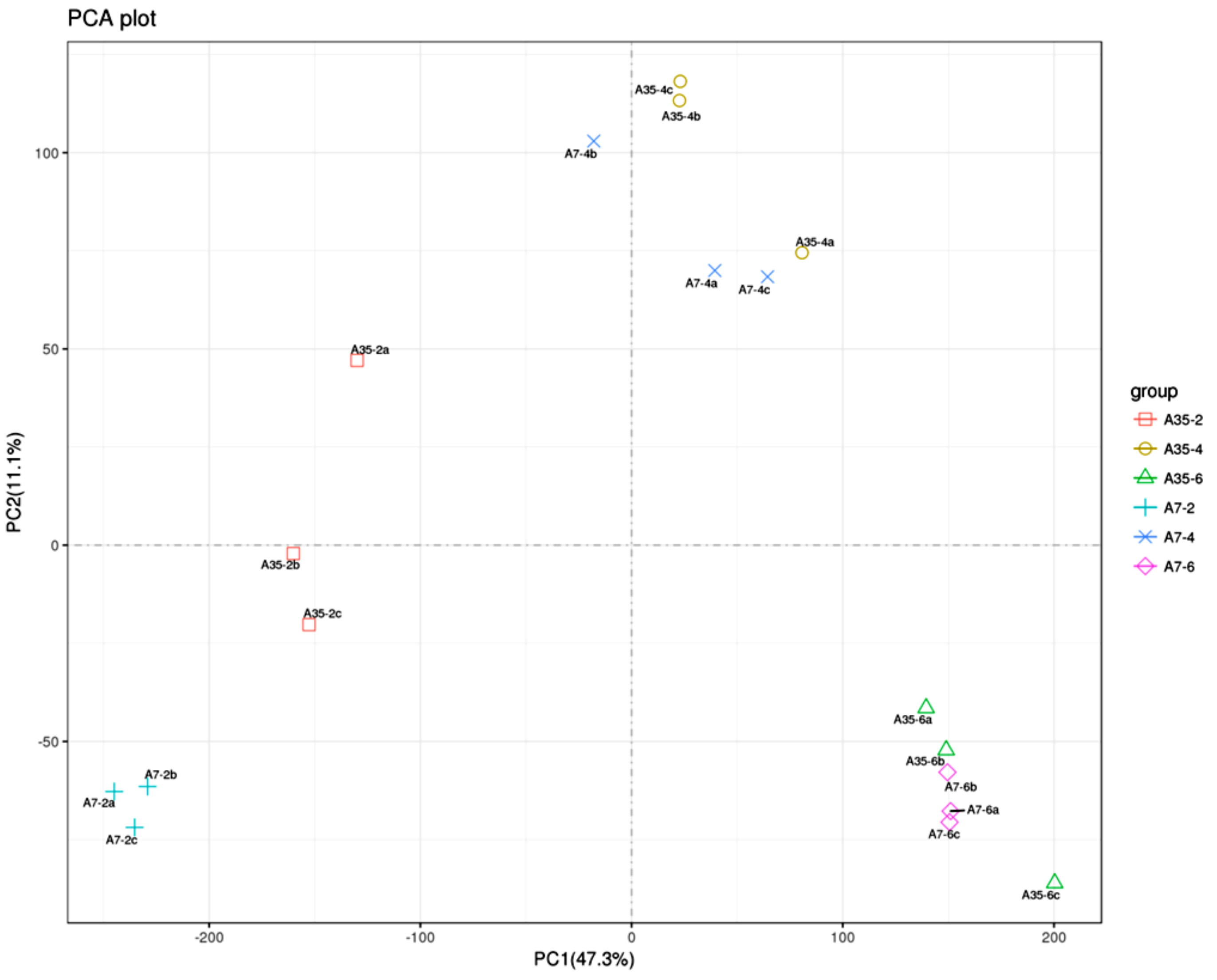

Figure S6. Clustering analysis of the DEGs. The tree was constructed with log10 (RPKM+1). Blue, white and red indicate high, intermediate and expression, respectively. The clustered genes were shown in the different color such as green, light blue and purple. 2 WAF, 4 WAF and 6 WAF seeds were labeled with A7-2,-4,-6 and A35-2, -4, -6. Each sample has three repetitions labeled a, b and c.

.

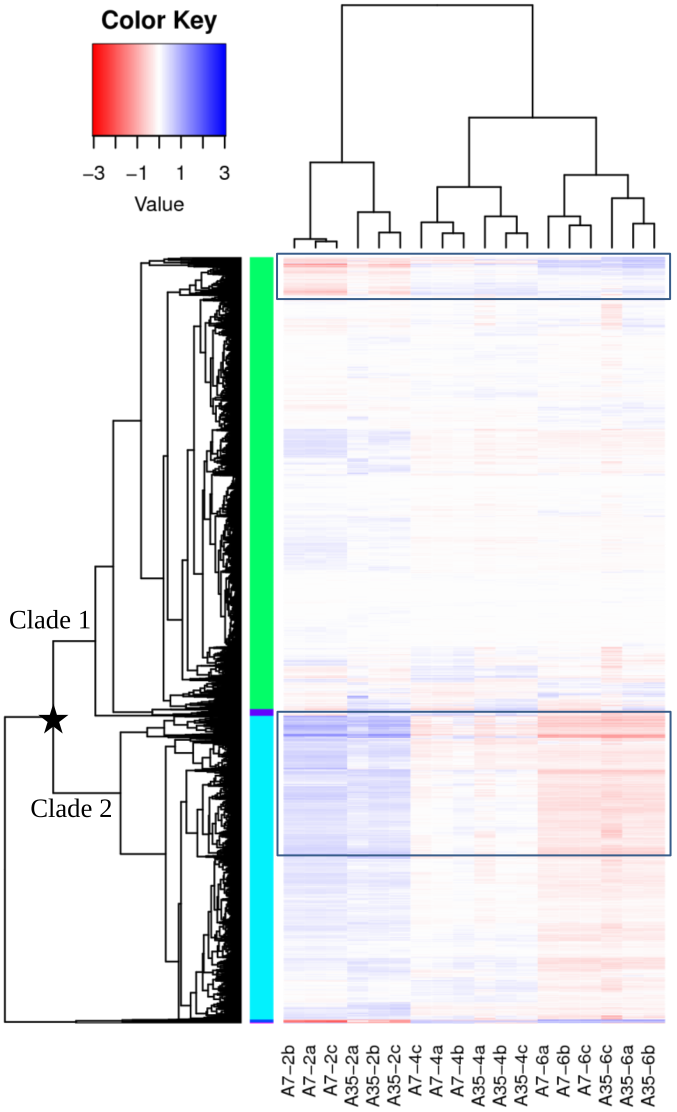

Figure S7. GO analysis of the DEGs. A1-A4: the enriched Go term in A35-2-VS-A7-2, A35-4-VS-A7-4, A35-6-VS-A7-6 and A35-VS-A7. B: the DEGs numbers in different comparative pairs. The functions related to molecular function, cellular component and biological process were shown in red, green and blue, respectively.

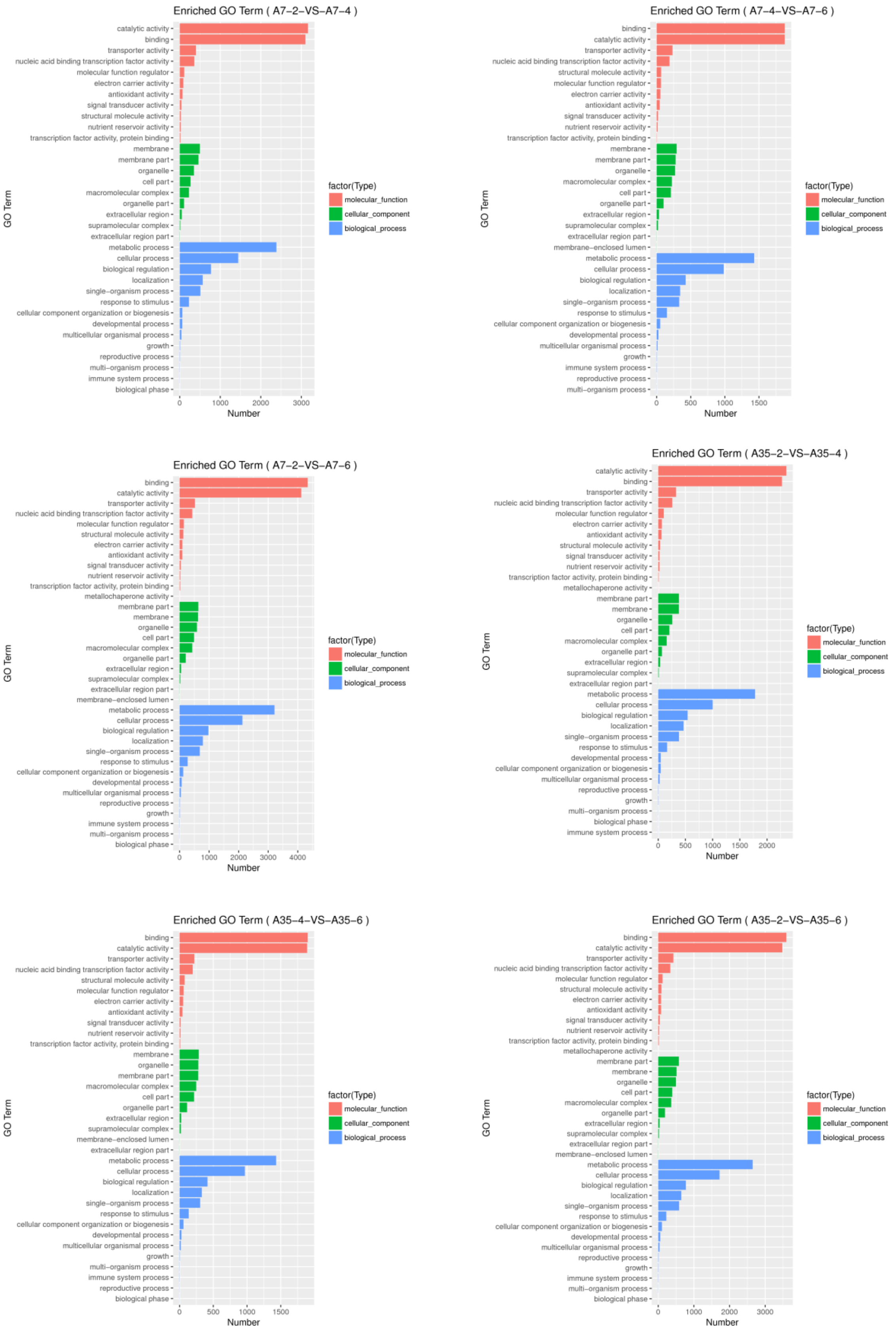

Figure S8.The fatty acids and amino acids metabolism pathway in KEGG database.

Fatty acid synthesis: ko00061

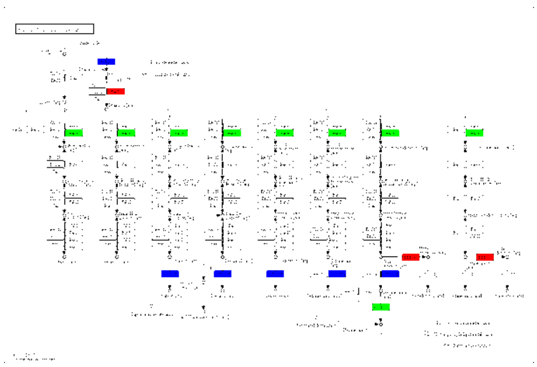

A7-2-VS-A7-4

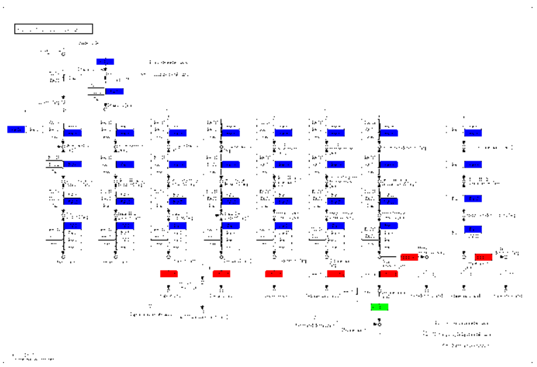

A35-4-VS-A35-6

Fatty acid elongation: ko00062

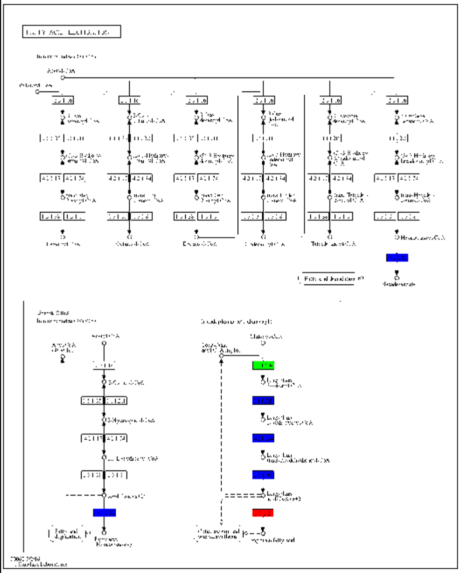

A7-2-VS-A7-6

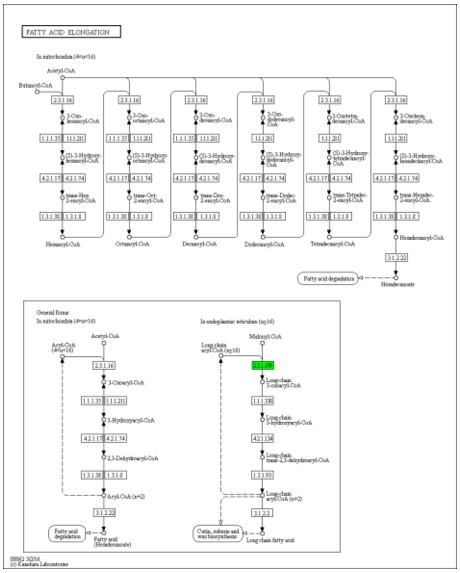

A35-2-VS-A7-2

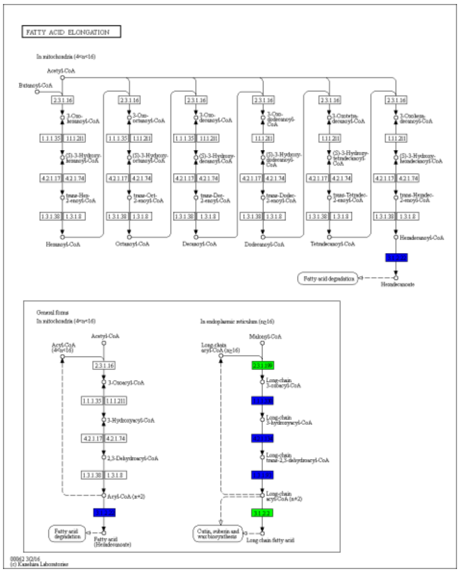

A35-2-VS-A35-6

Fatty acid degradation: ko00071

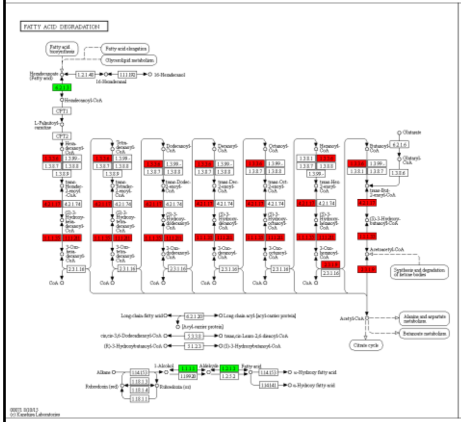

A7-2-VS-A7-4

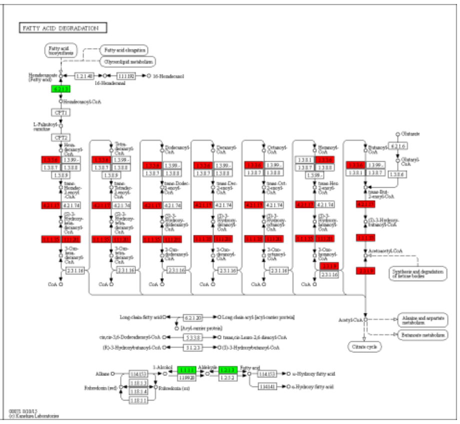

A7-2-VS-A7-6

Figure S9. The expression trends of asparaginase enzyme family in different comparison pairs. The Neighbor joining tree was constructed with amino acids sequences. The red, green and gray boxes represented their expression were down-regulated, up-regulated and not changed, respectively.

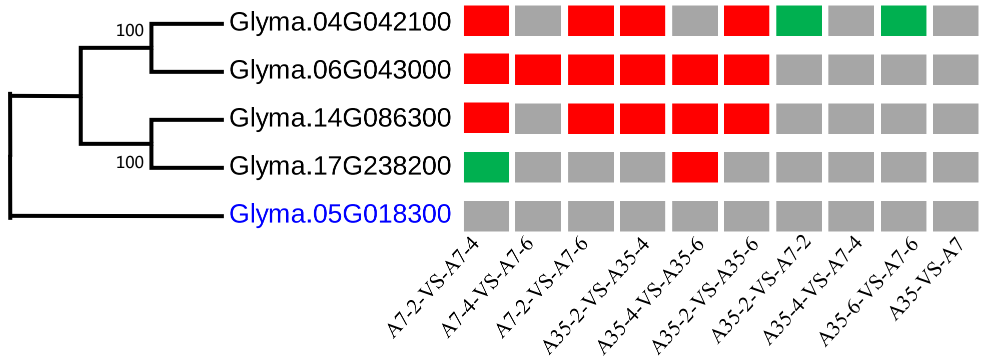

Supplement: Supplemental Information 2 — Figure S1. The saturation curves of all the samples in A7 and A35. Figure S2. The evenness curves of samples in A7 and A35. Figure S3. The correlation analysis of samples in A7 and A35. Figure S4. The principal component analysis (PCA) analysis of samples in A7 and A35. Figure S5. Clustering analysis of the DEGs. The tree was constructed with log10 (RPKM+1). Blue, white and red indicate high, intermediate and expression, respectively. The clustered genes were shown in the different color such as green, light blue and purple. 2 WAF, 4 WAF and 6 WAF seeds were labeled with A7-2,-4,-6 and A35-2, -4, -6. Each sample has three repetitions labeled (A), (B) and (C). Figure S6. GO analysis of the DEGs. (A1)–(A4): the enriched Go term in A35-2-VS-A7-2, A35-4-VS-A7-4, A35-6-VS-A7-6 and A35-VS-A7. (B): the DEGs numbers in different comparative pairs. The functions related to molecular function, cellular component and biological process were shown in red, green and blue, respectively. Figure S7. The fatty acids and amino acids metabolism pathway in KEGG database. Figure S8. The expression trends of asparaginase enzyme family in different comparison pairs. The Neighbor joining tree was constructed with amino acids sequences. The red, green and gray boxes represented their expression were down-regulated, up-regulated and not changed, respectively. [file peerj-09-10772-s002.pdf]
