# Supplementary material for: Comparative transcriptome analysis during seeds development between two soybean cultivars
Source: PeerJ. 2021 Mar 1;9:e10772. doi: 10.7717/peerj.10772 (PMC7931715; doi:10.7717/peerj.10772)
Supplement: Supplemental Information 3 [file peerj-09-10772-s003.pdf]

转录组（有参）生物信息分析结题报告

Customer:赵嫚 **Contract No:80-89030164**
BI:陶春梅 **Email:NGS.Service@genewiz.com** **Date:2017-12-20**

PDF Methods Software

一、实验流程

转录组测序实验流程包括RNA提取、RNA样品质量检测、文库构建、文库纯化、文库检测、文库定量、测序簇的生成以及上机测序。每一个环节都会对数据质量和数量产生影响，而数据质量又会直接影响后续信息分析的结果，为了保证源头数据的准确性与可靠性，我们对每一步实验过程都进行严格质控，检测合格后，把不同文库按照有效浓度及目标下机数据量的需求混样后进行Illumina HiSeq测序。流程图如下：

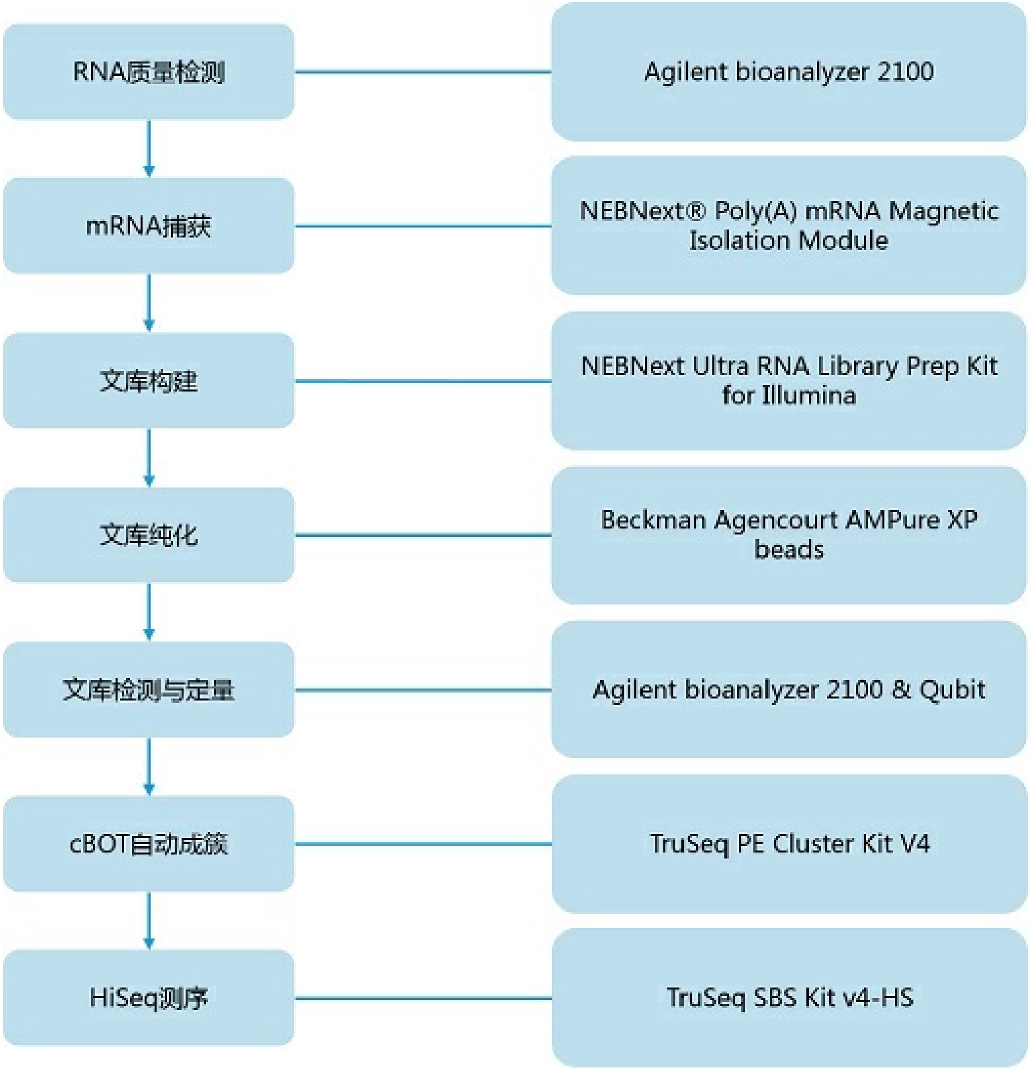

Figure 一.1 转录组实验流程

二、生物信息分析流程

转录组是特定细胞在某一功能状态下所能转录出来的所有RNA的总和，包括编码的mRNA和非编码RNA。转录组测序（Transcriptome sequencing）是基于Illumina HiSeq测序平台，研究特定组织或细胞在某个时期转录出来的所有mRNA，转录组研究是基因功能及结构研究的基础和出发点，通过新一代高通量测序，能够全面快速地获得某一物种特定组织或器官在某一状态下的几乎所有转录本序列信息，已广泛应用于基础研究、临床诊断和药物研发等领域。

获得原始测序数据（Pass Filter Data）后，通过如下流程进行生物信息分析：

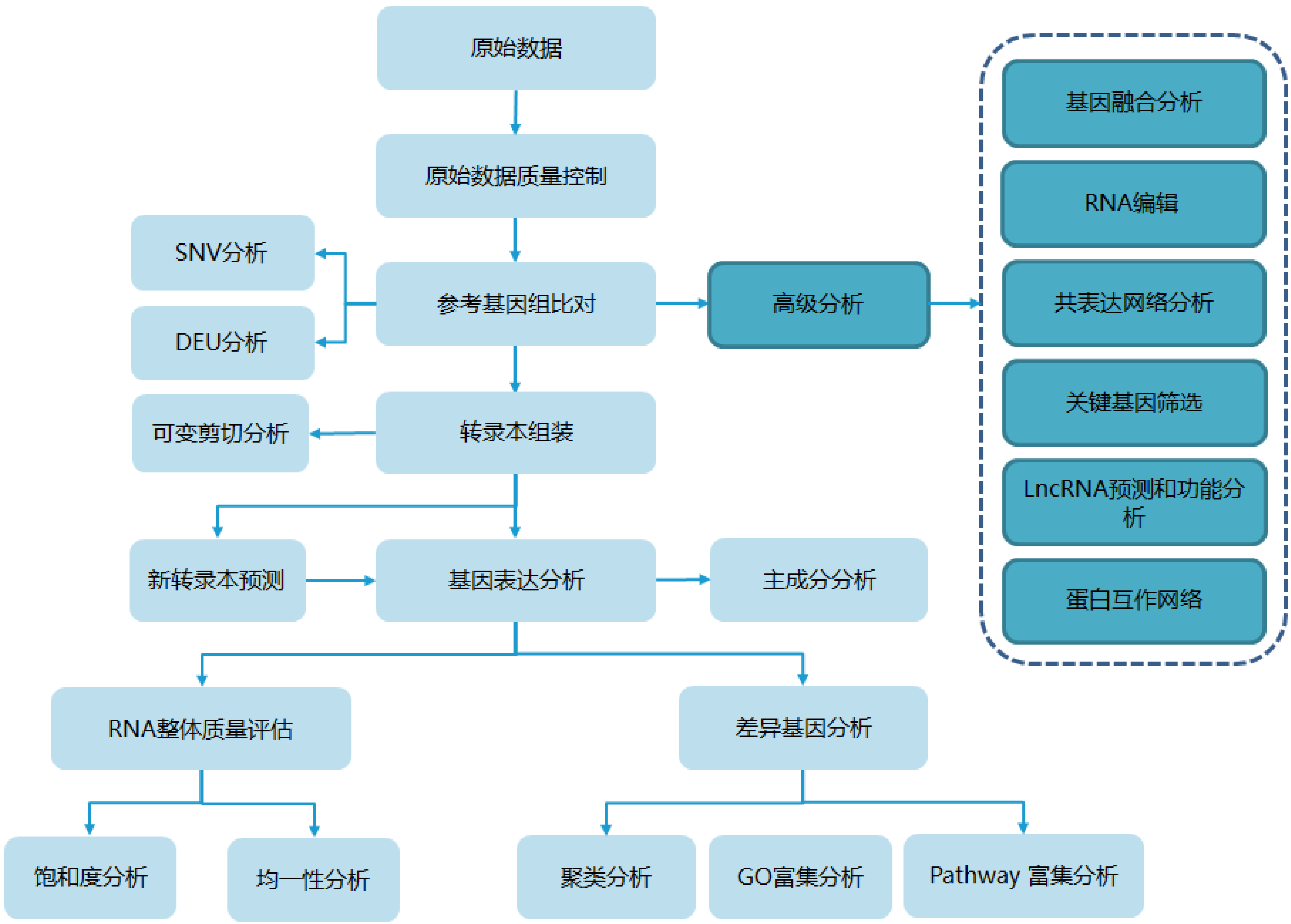

Figure 二.1 转录组数据分析流程

三、生物信息分析结果

1 原始序列数据

*.fq

本次实验样本及分组信息:

Table 1.1 Sample list.

SampleName	SampleName	SampleName	SampleName
A35-2a	A35-2b	A35-2c	A35-4a
A35-4b	A35-4c	A35-6a	A35-6b
A35-6c	A7-2a	A7-2b	A7-2c
A7-4a	A7-4b	A7-4c	A7-6a
A7-6b	A7-6c		

Table 1.2 Group information.

Control Group	Sample List	Experimental Group	Sample List
A7-2	A7-2a,A7-2b,A7-2c	A7-4	A7-4a,A7-4b,A7-4c
A7-2	A7-2a,A7-2b,A7-2c	A7-6	A7-6a,A7-6b,A7-6c
A7-4	A7-4a,A7-4b,A7-4c	A7-6	A7-6a,A7-6b,A7-6c
A35-2	A35-2a,A35-2b,A35-2c	A35-4	A35-4a,A35-4b,A35-4c
A35-2	A35-2a,A35-2b,A35-2c	A35-6	A35-6a,A35-6b,A35-6c
A35-4	A35-4a,A35-4b,A35-4c	A35-6	A35-6a,A35-6b,A35-6c
A35-2	A35-2a,A35-2b,A35-2c	A7-2	A7-2a,A7-2b,A7-2c
A35-4	A35-4a,A35-4b,A35-4c	A7-4	A7-4a,A7-4b,A7-4c
A35-6	A35-6a,A35-6b,A35-6c	A7-6	A7-6a,A7-6b,A7-6c
A35	A35-2a,A35-2b,A35-2c,A35-4a,A35-4b,A35-4c,A35-6a,A35-6b,A35-6c	A7	A7-2a,A7-2b,A7-2c,A7-4a,A7-4b,A7-4c,A7-6a,A7-6b,A7-6c

列名解释：

(1) Control Group 对照组名称
(2) Sample List 对照组样本列表
(3) Experimental Group 实验组名称
(4) Sample List 实验组样本列表

对测序结果原始图像数据利用软件Bcl2fastq (v2.17.1.14)进行图像碱基识别（Base Calling），初步质量分析（在测序过程中，Illumina内置软件根据每个测序片段，即read，前25个碱基的质量决定该read是保留还是舍弃），得到原始测序数据（Pass Filter Data），结果以 FASTQ 文件格式存储，其中包含测序序列信息（FASTQ格式第二行）及与其对应的测序质量信息（FASTQ格式第四行）。

FASTQ格式文件中每个read由四行描述，如下：

```
@GWZHISEQ01:289:C3Y96ACXX:6:1101:1704:2425 1:N:0:GGCTAC
GCTCTTTGCCCTTCTCGTCGAAAATTGTCTCCTCATTCGAAACTTCTCTGT
+
@@CFFFDEHHHHFIJJJ@FHGIIIEHIIJBHHHIJJEGIIJJIGHIGHCCF
```

每个序列共有4行，第1行和第3行是序列名称（有的fq文件为了节省存储空间会省略第三行“＋”后面的序列名称），由测序仪产生；第2行是序列；第4行是序列的测序质量，每个字符对应第2行每个碱基，第四行每个字符对应的ASCII值减去33，即为该碱基的测序质量值，比如@对应的ASCII值为64，那么其对应的碱基质量值是31。从Illumina GA Pipeline v1.8开始（目前为v1.9），碱基质量值范围为0到41。

Table 1.3 illumina 测序标识符详细信息。

Type	Description
GWZHISEQ01	Unique instrument name
289	Run ID
C3Y96ACXX	Flowcell ID
6	Flowcell lane
1101	Tile number within the flowcell lane
1704	'x'-coordinate of the cluster within the tile
2424	'y'-coordinate of the cluster within the tile
1	Member of a pair, 1 or 2 (paired-end or mate-pair reads only)
N	Y if the read fails filter (read is bad), N otherwise
0	0 when none of the control bits are on, otherwise it is an even number
GGCTAC	Index sequence

2 测序数据质量评估

rawread_stat.txt
cleanreads_stat.txt
Sample_R1/2_(raw/trim)_type(GC/base/seq).png

2.1 测序数据质量分析

测序碱基质量受测序仪本身、测序试剂以及样品等多个因素共同影响，通常测序序列5’端前几个碱基错误率较高，随着测序序列长度的延伸，3’端碱基错误率会不断升高，这是高通量测序技术特点决定的(Erlich and Mitra, 2008; Jiang et al.)。 前6 个碱基的位置也会发生较高的测序错误率，而这个长度也正好等于在RNA-seq 建库过程中反转录所需要的随机引物的长度。所以推测前6 个碱基测序错误率较高的原因为随机引物和RNA 模版的不完全结合(Jiang et al.)。测序错误率分布检查用于检测在测序长度范围内，有无异常的碱基位置存在高错误率，比如中间位置的碱基测序错误率显著高于其他位置。一般情况下，每个碱基位置的测序错误率都应该低于0.5%。如果测序错误率用e表示，illumina HiSeqTM/MiSeq的碱基质量值用Qphred表示，则有下列关系：

公式一： $Q_{phred} = -10\log_{10}(e)$

Table 2.1.1 illumina Bcl2fastq碱基识别与Phred分值之间的简明对应关系

Phred Quality Score	Probability of Incorrect Base Call	Base Call Accuracy
10	1 in 10	90%
20	1 in 100	99%
30	1 in 1000	99.9%
40	1 in 10000	99.99%
50	1 in 100000	100%

测序数据质量评估采用软件FastQC（v0.10.1）进行分析。

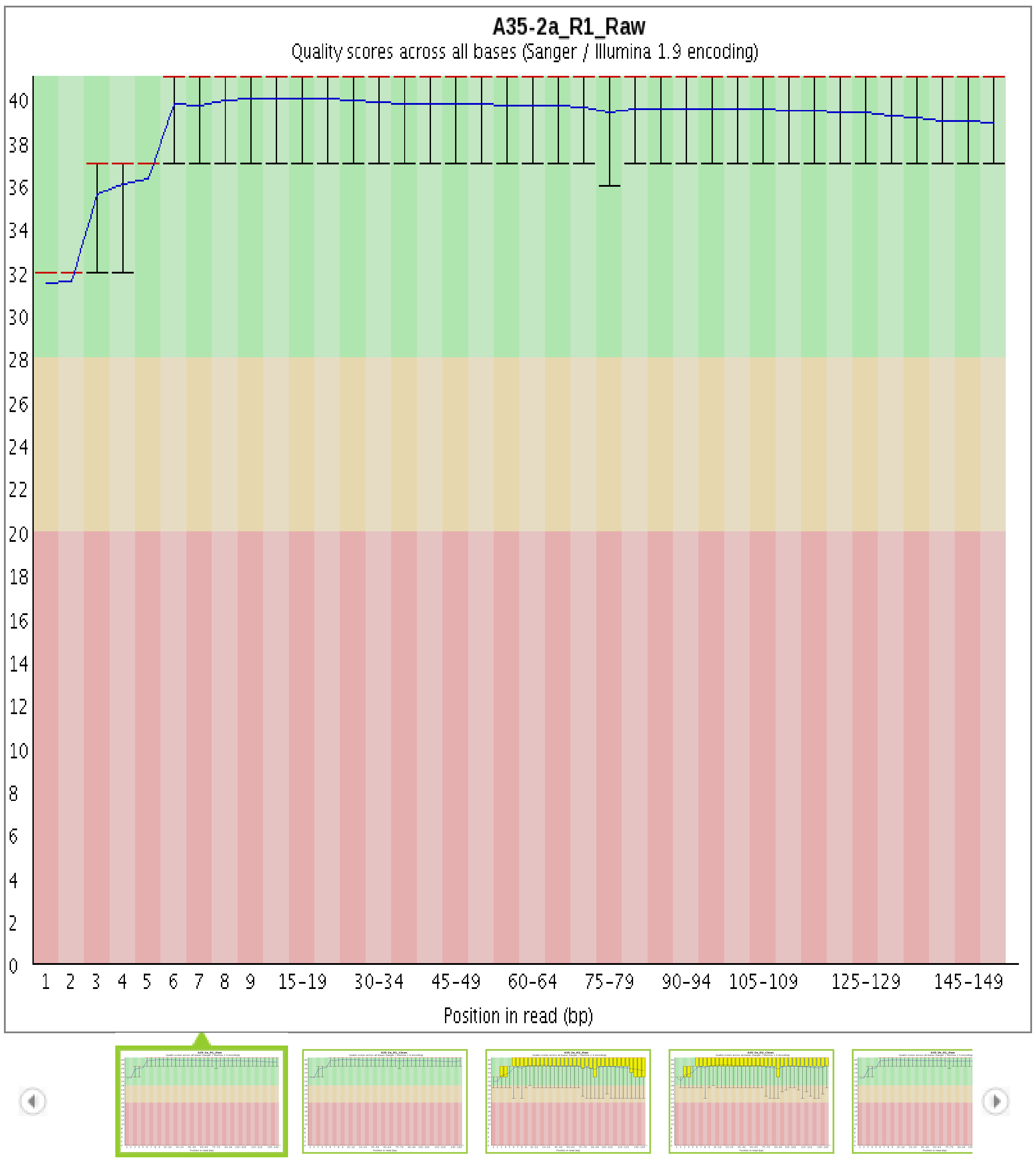

Figure 2.1.1 样品碱基位置质量分数分布情况展示，其中横坐标为每条reads的相对碱基位点，纵坐标代表测序质量分数，分数越高碱基越可信，一般碱基的质量值为13,错误率为5%，质量值为20错误率为1%，质量值为30的错误率为0.1%。

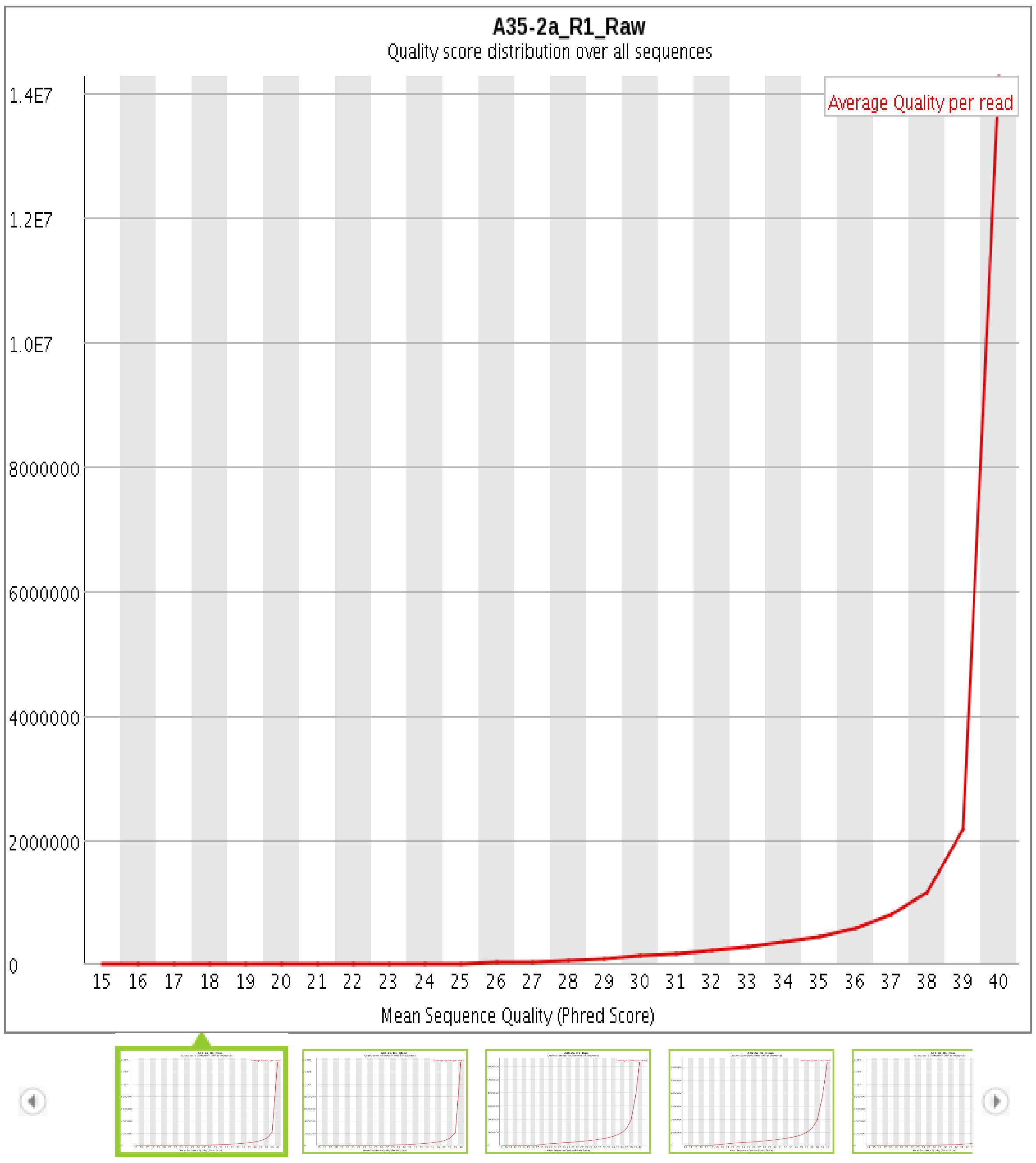

Figure 2.1.2 样品碱基序列平均质量分布情况，横坐标为序列平均碱基质量值，纵坐标代表序列数量，从图上可以看到绝大部分碱基序列的平均质量值峰值，该值一般大于30，可以判断序列质量较好。

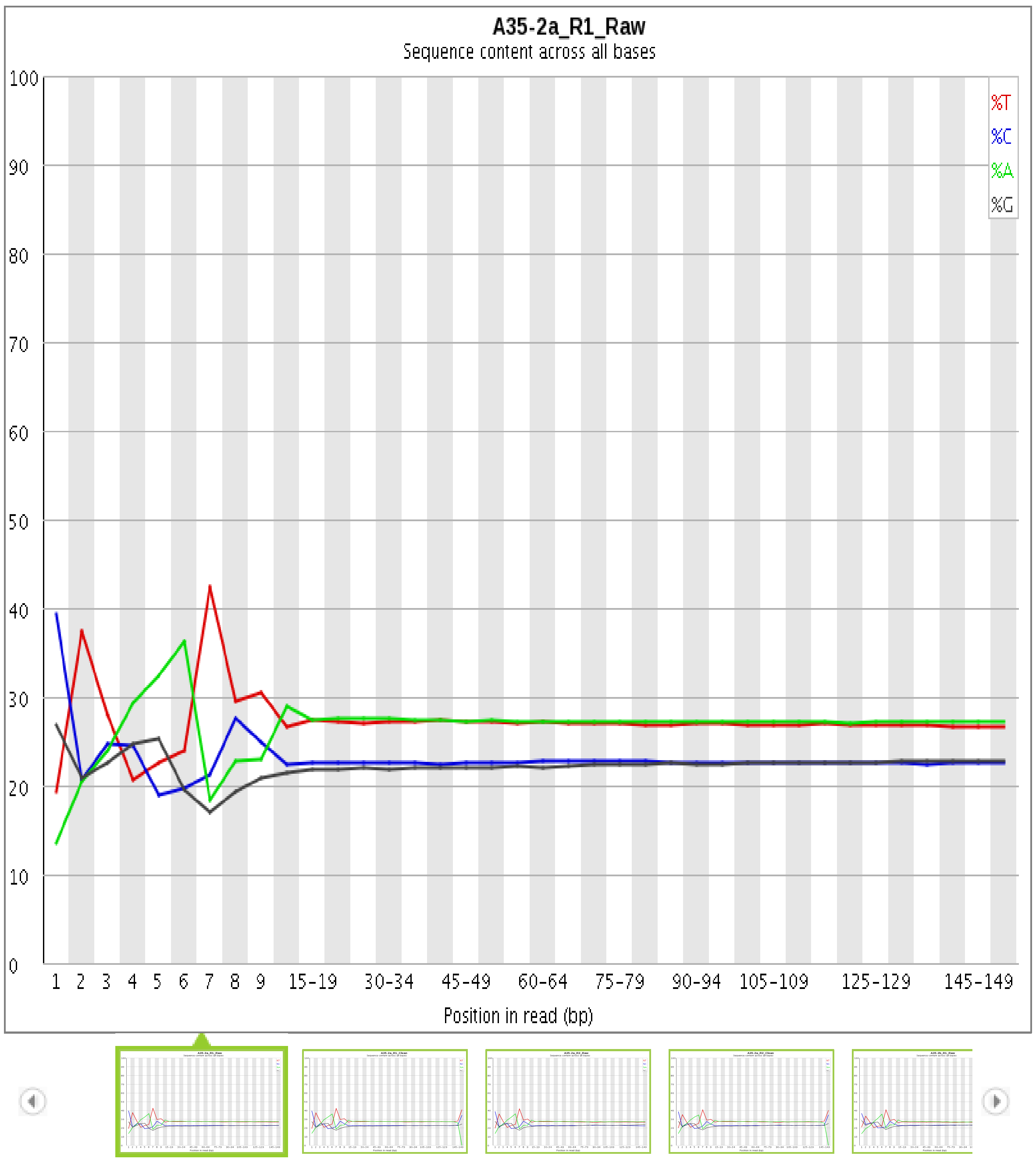

Figure 2.1.3 GC含量分布检查用于检测有无AT、GC 分离现象，横坐标为reads的碱基位置，纵坐标为单碱基所占的比例；不同颜色代表不同的碱基类型。

2.2 测序数据过滤

在测序过程中，有少量reads被测到接头序列，或者由于测序长度过大后导致reads的3端bases质量过低的现象，这些数据会对后续的分析结果产生负面的影响。因此，需要对原始数据进行预处理，对低质量数据进行过滤，去除污染及接头序列。

软件：Cutadapt（version 1.9.1）

方法说明：

(1) 去除接头(adapter)序列；
(2) 去除5'或3'末端质量值低于20或者含N的碱基；
(3) 去除trim后reads长度低于75bp的序列。

各样品测序原始数据统计如下表：

Table 2.2.1 测序原始数据质量统计

Sample	length	Reads	Bases	Q20 (%)	Q30 (%)	GC (%)	N (ppm)
A35-2a	150.00	42016114	6302417100	96.05	90.97	45.55	86.98
A35-2b	150.00	43074524	6461178600	95.85	90.53	46.57	77.89
A35-2c	150.00	51779906	7766985900	96.00	90.83	47.37	90.01
A35-4a	150.00	40314804	6047220600	97.26	93.30	47.13	27.36
A35-4b	150.00	40607384	6091107600	96.29	91.39	45.69	88.01
A35-4c	150.00	64859306	9728895900	97.13	93.02	45.41	42.90
A35-6a	150.00	63415536	9512330400	97.06	92.84	47.11	42.83
A35-6b	150.00	35975622	5396343300	96.22	91.31	45.71	11.42
A35-6c	150.00	40018416	6002762400	97.03	92.81	48.62	28.30
A7-2a	150.00	61643564	9246534600	96.16	90.90	44.43	4.60
A7-2b	150.00	53099460	7964919000	96.19	90.96	45.04	4.63
A7-2c	150.00	66399164	9959874600	96.40	91.33	45.01	4.52
A7-4a	150.00	53880184	8082027600	96.00	90.58	46.06	4.55
A7-4b	150.00	47427190	7114078500	96.17	91.11	45.61	74.71
A7-4c	150.00	46128656	6919298400	96.22	91.26	45.86	86.01
A7-6a	150.00	40886784	6133017600	97.18	93.13	46.48	20.30
A7-6b	150.00	44831112	6724666800	95.89	90.64	46.24	87.56
A7-6c	150.00	48869158	7330373700	96.32	91.34	46.41	53.52

各样品测序数据过滤后统计如下表：

Table 2.2.2 过滤后数据质量统计

Sample	length	Reads	Bases	Q20 (%)	Q30 (%)	GC (%)	N (ppm)
A35-2a	148.45	41668866	6185685718	96.49	91.57	45.56	27.00
A35-2b	147.93	42685424	6314286134	96.32	91.18	46.56	25.10
A35-2c	148.25	51335092	7610571053	96.46	91.46	47.37	28.35
A35-4a	148.78	40149578	5973616868	97.53	93.67	47.18	15.24
A35-4b	148.36	40312024	5980721139	96.68	91.93	45.70	28.49
A35-4c	148.67	64609700	9605749123	97.39	93.38	45.44	39.23
A35-6a	148.41	63135762	9370242608	97.35	93.23	47.14	39.04
A35-6b	148.81	35676040	5309056985	96.70	91.96	45.72	4.49
A35-6c	148.85	39818710	5926988533	97.34	93.23	48.68	15.93
A7-2a	148.37	61149382	9072735440	96.71	91.66	44.43	3.29
A7-2b	148.29	52685944	7812842489	96.74	91.72	45.04	3.34
A7-2c	148.42	65943616	9787577914	96.90	92.01	45.02	3.25
A7-4a	148.35	53413584	7923937503	96.59	91.39	46.07	3.25
A7-4b	148.67	47088076	7000791610	96.57	91.66	45.63	23.62
A7-4c	148.67	45787236	6806968424	96.62	91.81	45.88	26.15
A7-6a	148.80	40729736	6060644045	97.43	93.48	46.53	11.89
A7-6b	148.44	44435570	6595796642	96.36	91.27	46.25	26.86
A7-6c	148.57	48520882	7208815045	96.70	91.86	46.43	17.57

列名解释：

(1) Sample: 测序样品名称。
(2) length: 平均长度。
(3) Reads: 测序reads数量。
(4) Bases: 总碱基数量。
(5) Q20、Q30：分别计算Phred数值大于20、30的碱基占总体碱基的百分比。
(6) GC%：计算碱基G和C的数量总和占总的碱基数量的百分比。
(7) N(ppm)：每百万碱基中N的数量。

3 参考序列比对分析

mapping_stat.txt
*.bam
*_dis.png
*_chr.png

3.1 clean data与参考基因组比对分析

将过滤后的测序Clean Data与参考基因组进行比对分析，选择合适的参考基因组对项目信息分析的成功至关重要，数据比对率一定程度可以反映实验测序样品与选择的参考基因组相似性关系。

采用Hisat2(v2.0.1)软件进行短reads的比对，默认参数。

参考基因组及注释文件

Table 3.1.1 Clean reads 比对到参考基因组上情况

Samples	Total reads	Total mapped	Multiple mapped	Uniquely mapped	Read1	Read2	Reads map to '+'	Reads map to '-'	Non_splice reads	Splice reads	Reads mapped in proper pairs
A35-2a	41668866	37590590 (90.2127%)	2666714 (6.39978%)	34923876 (83.8129%)	18198169	16725707	17451949	17471927	20964773	13959103	32737650
A35-2b	42685424	36303070 (85.0479%)	4740563 (11.1058%)	31562507 (73.9421%)	16459954	15102553	15763743	15798764	19847405	11715102	29529136
A35-2c	51335092	43466985 (84.673%)	5956991 (11.6041%)	37509994 (73.0689%)	19532986	17977008	18730389	18779605	23043912	14466082	35151510
A35-4a	40149578	36658511 (91.3048%)	4420508 (11.0101%)	32238003 (80.2947%)	16410570	15827433	16082467	16155536	20364023	11873980	30501262
A35-4b	40312024	36527997 (90.6132%)	3917213 (9.71723%)	32610784 (80.8959%)	16940206	15670578	16293362	16317422	20937920	11672864	30586404
A35-4c	64609700	59941034 (92.774%)	6645450 (10.2855%)	53295584 (82.4885%)	27108395	26187189	26627351	26668233	33630622	19664962	51002774
A35-6a	63135762	54364474 (86.1073%)	11212218 (17.7589%)	43152256 (68.3484%)	21951710	21200546	21534219	21618037	29343867	13808389	41074662
A35-6b	35676040	32933586 (92.3129%)	4582496 (12.8447%)	28351090 (79.4682%)	14579527	13771563	14127997	14223093	19087618	9263472	26689824
A35-6c	39818710	36000737 (90.4116%)	5775073 (14.5034%)	30225664 (75.9082%)	15407636	14818028	15056942	15168722	21079442	9146222	28417272
A7-2a	61149382	56477437 (92.3598%)	3154377 (5.15848%)	53323060 (87.2013%)	27304861	26018199	26632611	26690449	33029822	20293238	50916418
A7-2b	52685944	47416555 (89.9985%)	4138614 (7.85525%)	43277941 (82.1432%)	22142043	21135898	21613568	21664373	27116443	16161498	41387844
A7-2c	65943616	60102169 (91.1418%)	4652240 (7.05488%)	55449929 (84.0869%)	28305023	27144906	27691887	27758042	34283114	21166815	53147404
A7-4a	53413584	49110730 (91.9443%)	6390438 (11.9641%)	42720292 (79.9802%)	21907077	20813215	21314900	21405392	27385106	15335186	40568056
A7-4b	47088076	42922569 (91.1538%)	4747228 (10.0816%)	38175341 (81.0722%)	19832223	18343118	19059894	19115447	23804083	14371258	35805320
A7-4c	45787236	41579551 (90.8104%)	5567452 (12.1594%)	36012099 (78.651%)	18732956	17279143	17966671	18045428	23433484	12578615	33684158
A7-6a	40729736	37631104 (92.3922%)	5893197 (14.469%)	31737907 (77.9232%)	16158300	15579607	15810987	15926920	21354591	10383316	30232328
A7-6b	44435570	40278198 (90.644%)	6990346 (15.7314%)	33287852 (74.9126%)	17387475	15900377	16593288	16694564	22328699	10959153	31003618
A7-6c	48520882	44381381 (91.4686%)	7628482 (15.7221%)	36752899 (75.7466%)	19006462	17746437	18319899	18433000	24204235	12548664	34558986

列名解释：

(1) Total reads：测序序列经过测序数据过滤后的数量统计(Clean data)。
(2) Total mapped：能定位到基因组上的测序序列的数量的统计；一般情况下，如果不存在污染并且参考基因组选择合适的情况下，这部分数据的百分比大于 70%。
(3) Multiple mapped：在参考序列上有多个比对位置的测序序列的数量统计；这部分数据的百分比一般会小于10%。
(4) Uniquely mapped：在参考序列上有唯一比对位置的测序序列的数量统计。
(5) Reads map to '+'，Reads map to '-'：测序序列比对到基因组上正链和负链的统计。
(6) Splice reads：(2)中，分段比对到两个外显子上的测序序列(也称为Junction reads)的统计，Non-splice reads为整段比对到外显子的将测序序列的统计，Splice reads的百分比取决于测序片段的长度。
(7) Reads mapped in proper pairs: 双端测序序列比对到染色体上合理的方向和位置。

3.2 Reads在参考基因组不同区域的分布情况

对Total mapped reads比对到基因组上的各个部分的情况进行统计，定位区域分为exon（外显子）、intron（内含子）和intergenic（基因间隔区域）。定位到Intron (内含子) 区域的测序序列可能是由于非成熟的mRNA 的污染或者基因组注释不完全导致的，而定位到Intergenic(基因间隔区域)的测序序列可能是因为基因组注释不完全以及背景噪音。

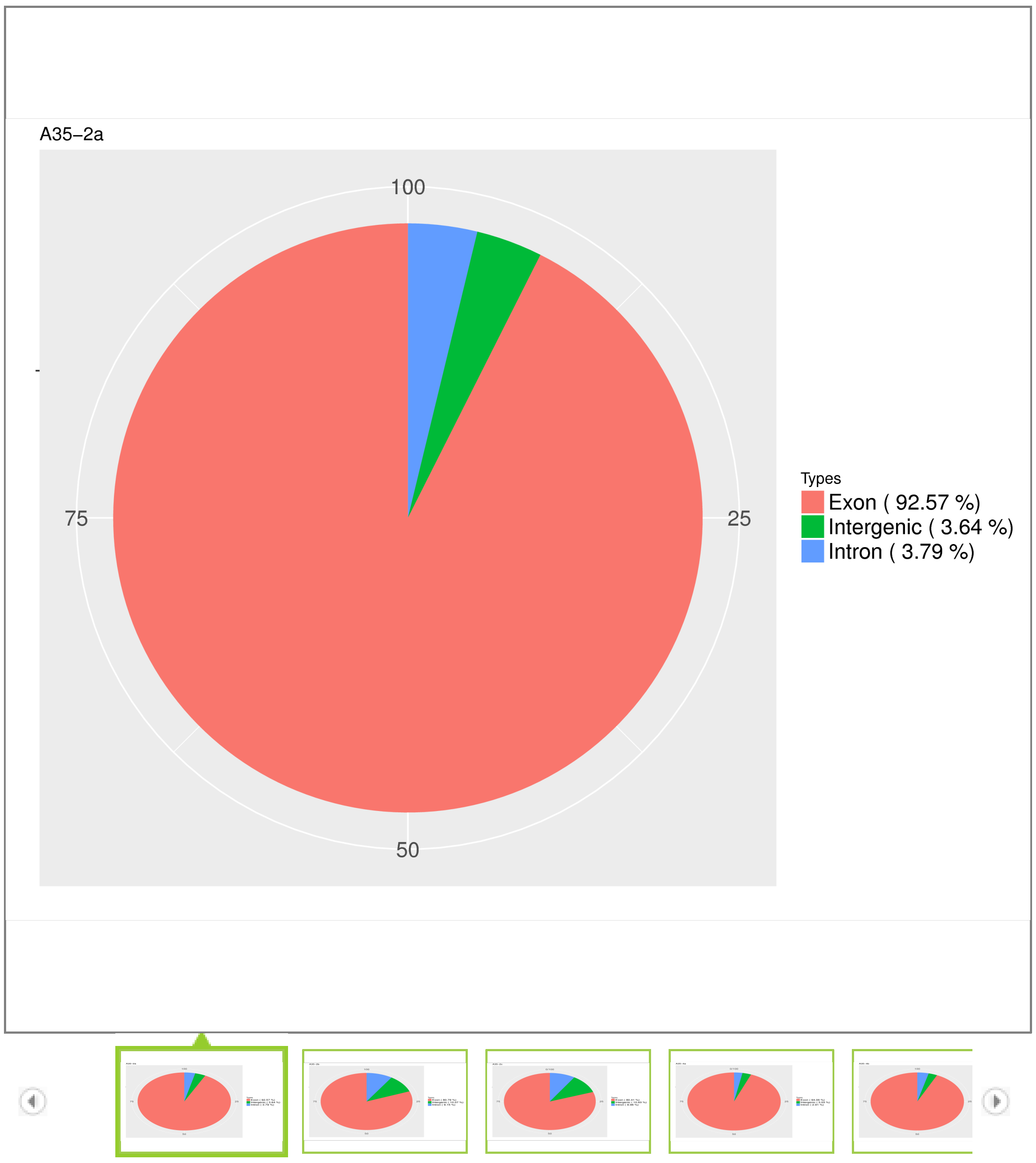

Figure 3.2.1 Reads在参考基因组不同区域的分布情况

3.3 Reads在染色体上的密度分布情况

对 Total mapped reads 比对到基因组上的各个染色体进行统计，如下图所示。具体作图的方法为计算窗口内部比对到碱基位置上的reads 数目，并计算其在染色体上的深度分布，并取log2值 。正常情况下，整个染色体长度越长，该染色体内部定位的reads 总数会越多(Marquez et al.)。从定位到染色体上的reads 数目与染色体长度的关系图中，可以更加直观看出测序的均匀度。

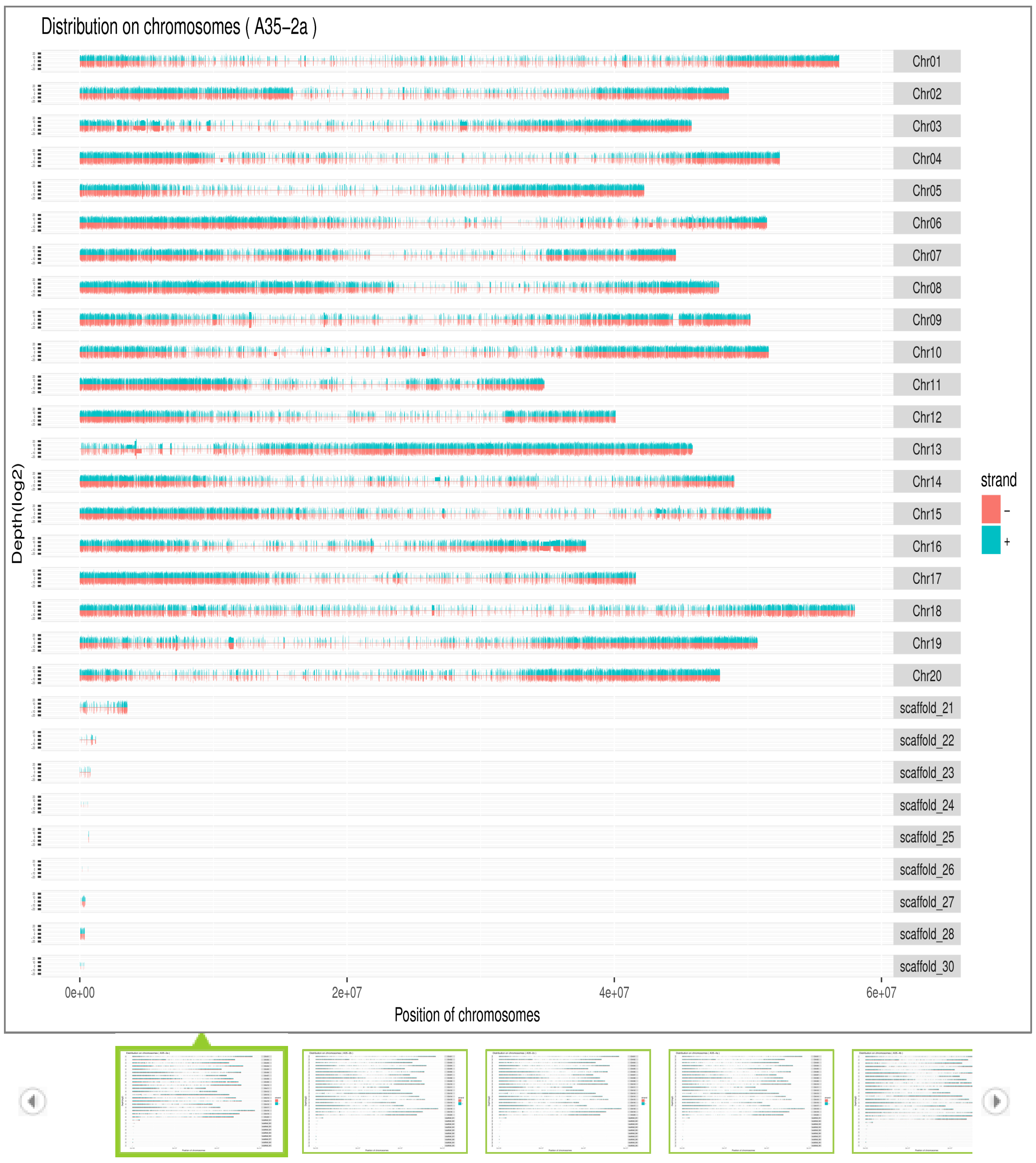

Figure 3.3.1 Reads在染色体上的密度分布图，纵坐标为序列在染色体上的深度分布并取log2值，横坐标为染色体的长度。

3.4 Reads比对结果可视化

我们提供 RNA-seq Reads 在基因组上比对结果的bam 格式文件，并推荐使用IGV (Integrative Genomics Viewer) 浏览器对bam 文件进行可视化浏览。IGV浏览器可在不同尺度上显示基因不同区域的丰度，以反映不同区域的转录水平。

IGV下载

IGV操作手册

IGV使用方法：

(1) 上传参考基因组方法：选择 Genomes>Load Genome From File，常见的模式生物，例如人的可以直接从下拉列表里选择参考基因组。不常见的物种染色体组，我们会提供染色体组的下载地址，客户可采取下载完毕后，从文件菜单中选择载入;
(2) 上传与基因上比对结果的bam 格式文件：选择File > Load From File，依次选择各样品的排序后bam文件导入即可;
(3) 在工具栏第二个条目选择要查看的参考基因，结果如下：

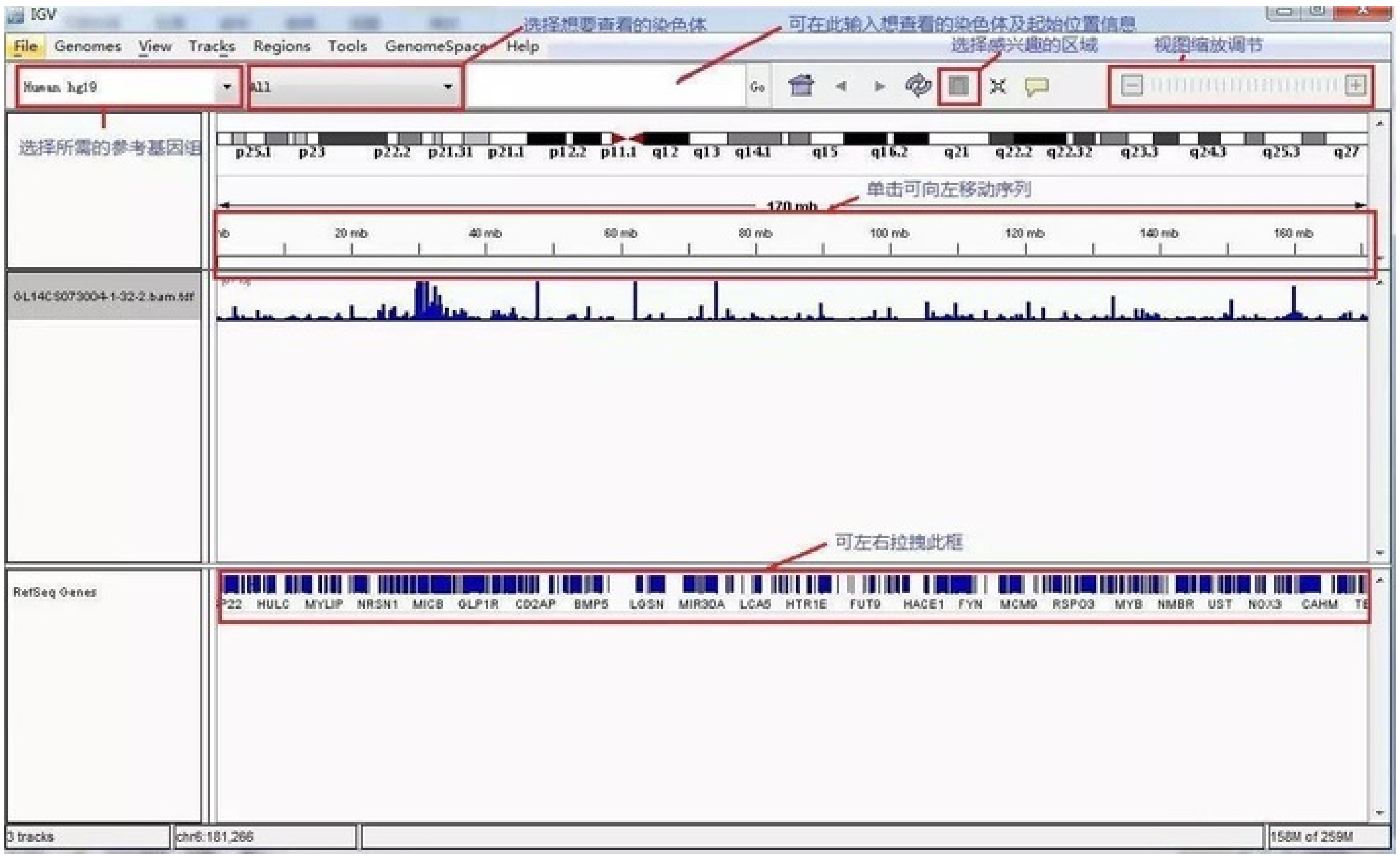

Figure 3.4.1 IGV浏览器界面

4 可变剪切分析

AS.png
*.anno.fpkm.xls

可变剪接使一个基因产生多个mRNA转录本，不同mRNA可能翻译成不同蛋白。因此，通过可变剪接一个基因可能产生多个蛋白，极大地增加了蛋白多样性（Black, 2003；Stamm, 2005；Lareau, 2004）。虽然已知可变剪接在真核生物中普遍存在，但我们可能仍低估了可变剪接的比例，最近，基于高通量测序的可变剪接研究在人（Pan, 2008；Wang, 2008；Sultan, 2008）、小鼠（Tang, 2009；Mortazavi, 2008）、拟南芥（Filichkin）中发现了很多新的可变剪接事件。

用 ASprofile（V1.0.4） 软件对StringTie(v1.0.4)(Nature Biotechnology 2015；Pertea M, et al.） 预测出的转录本进行可变剪切事件分别进行分类和表达量统计。ASprofile 中的可变剪切事件分类如下图所示：

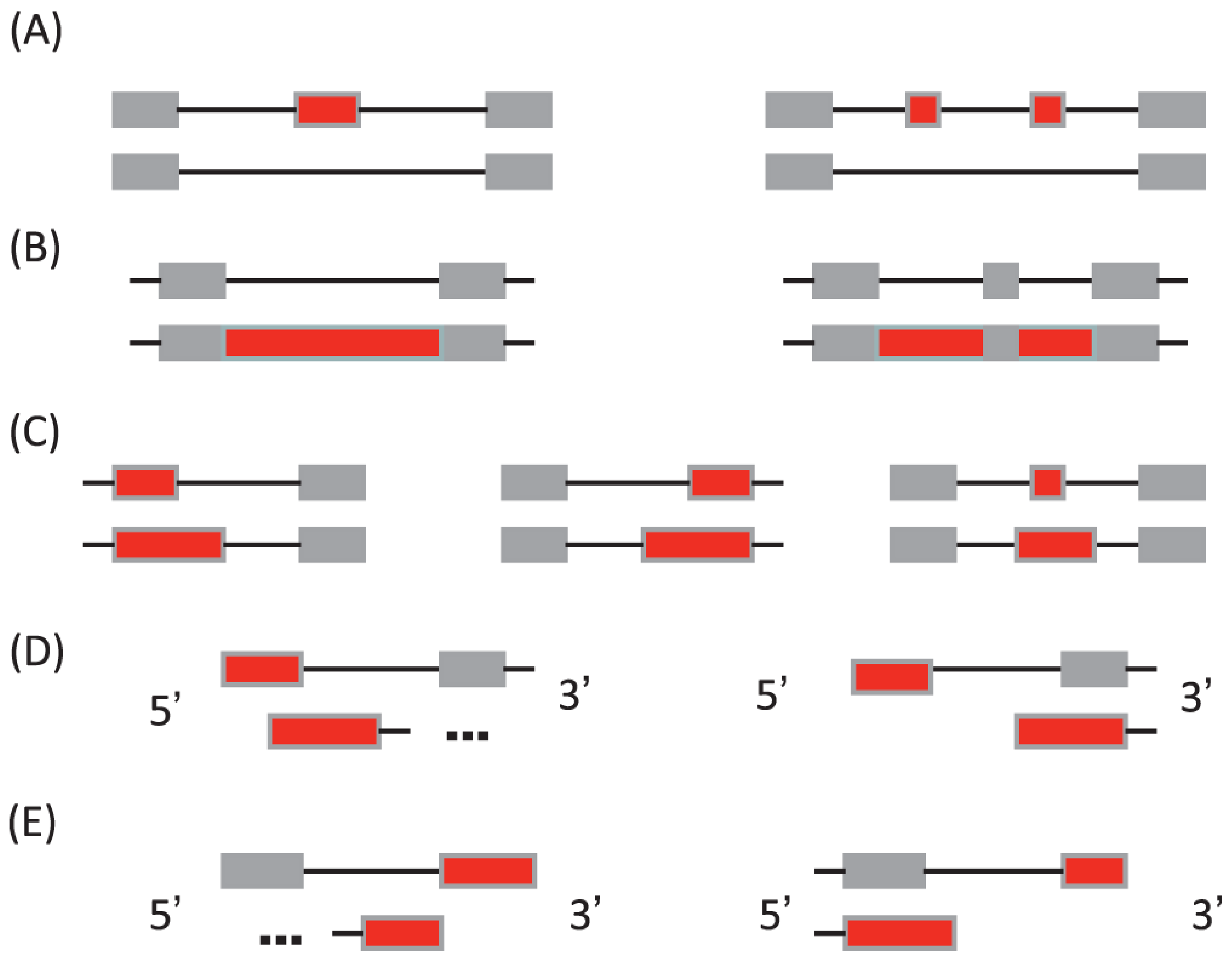

Figure 4.1 基本可变剪切事件：(A) SKIP，MSKIP; (B) IR，MIR; (C) AE; (D) TSS;(E) TTS. 可变剪切部分被标记为红色。

(A) SKIP: Skipped exon (SKIP_ON,SKIP_OFF pair) 单外显子跳跃
XSKIP: Approximate SKIP (XSKIP_ON,XSKIP_OFF pair) 单外显子跳跃（模糊边界）
MSKIP: Multi-exon SKIP (MSKIP_ON,MSKIP_OFF pair) 多外显子跳跃
XMSKIP: Approximate MSKIP (XMSKIP_ON,XMSKIP_OFF pair) 多外显子跳跃（模糊边界）
(B) IR: Intron retention (IR_ON, IR_OFF pair) 单内含子滞留
XIR: Approximate IR (XIR_ON, XIR_OFF pair) 单内含子滞留（模糊边界）
MIR: Multi-IR (MIR_ON, MIR_OFF pair) 多内含子滞留
XMIR: Approximate MIR (XMIR_ON, XMIR_OFF pair) 多内含子滞留（模糊边界）
(C) AE: Alternative exon ends (5', 3', or both) 可变 5'或3'端剪切
XAE: Approximate AE 可变 5'或3'端剪切（模糊边界）
(D) TSS: Alternative 5' first exon (transcription start site) 第一个外显子可变剪切
(E) TTS: Alternative 3' last exon (transcription terminal site) 最后一个外显子可变剪切

4.1 可变剪切事件分类和数量统计

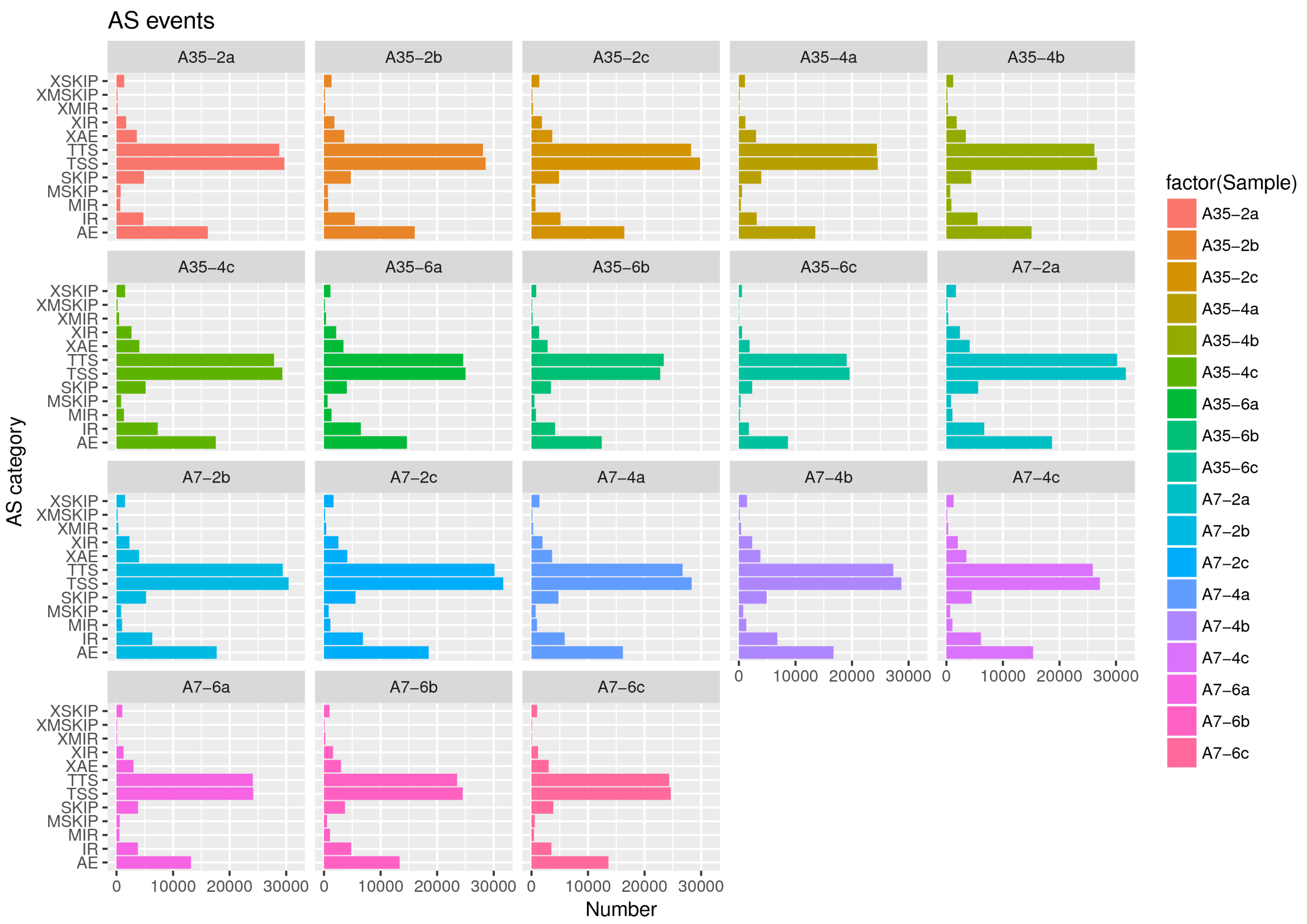

Figure 4.1.1 可变剪切种类与数目统计图;纵坐标为可变剪切事件的分类缩写；横坐标为该种事件下可变剪切的数量；不同样品用不同子图和颜色区分。

4.2 可变剪切事件结构和表达量统计

Table 4.2.1 AS结构和表达量统计（截取部分数据展示，详见*.anno.fpkm.xls）

event_id	event_type	gene_id	chrom	event_start	event_end	event_pattern	strand	fpkm	ref_id
1000002	TTS	XLOC_000001	Chr01	63369	63419	63369	+	0.5453940000	Glyma.01G000200
1000007	TSS	XLOC_000004	Chr01	116094	118482	118482	+	1.4703410000	Glyma.01G000600
1000009	TTS	XLOC_000004	Chr01	127392	127845	127392	+	1.4703410000	Glyma.01G000600
1000011	SKIP_OFF	XLOC_000004	Chr01	125587	125837	118482,127392	+	1.4703410000	Glyma.01G000600
1000014	TSS	XLOC_000005	Chr01	143467	143547	143547	+	1.4104680000	Glyma.01G000900

(1) event_id: AS事件编号
(2) event_type: AS事件类型 (TSS, TTS, SKIP_{ON,OFF}, XSKIP_{ON,OFF}, MSKIP_{ON,OFF}, XMSKIP_{ON,OFF}, IR_{ON,OFF}, XIR_{ON,OFF}, AE, XAE)
(3) gene_id: cuffmerge组装结果中的基因编号
(4) chrom: 染色体编号
(5) event_start: AS事件起始位置
(6) event_end: AS事件结束位置
(7) event_pattern: AS事件特征 (for TSS, TTS - inside boundary of alternative marginal exon; for *SKIP_ON,the coordinates of the skipped exon(s); for *SKIP_OFF, the coordinates of the enclosing introns; for *IR_ON, the end coordinates of the long, intron-containing exon; for *IR_OFF, the listing of coordinates of all the exons along the path containing the retained intron; for *AE, the coordinates of the exon variant)
(8) strand: 基因正负链信息
(9) fpkm: 此AS类型该基因表达量
(10) ref_id: 此基因在参考注释文件中的编号

5 新转录本预测

*_comp.combined.gtf
*_novel.xls

现有数据库中对转录本的注释可能还不全面，通过高通量测序我们能检测到新的转录本（Mortazavi, 2008）。将所有测序 reads 数据的基因组比对结果放到一起，用 StringTie(v1.0.4)（Nature Biotechnology 2015；Pertea M, et al.） 进行组装，然后用Cuffcompare 和已知的基因模型 (*.gtf)进行比较，可以：

(1) 发现新的未知基因（相对于原有基因注释文件）；
(2) 发现已知基因新的外显子区域；
(3) 对已知基因的起始和终止位置进行优化。

新基因和新外显子区域预测结果为GTF 格式的注释文件。GTF 格式的详细说明可参考: GTF格式。

Table 5.1 新转录本结构注释结果（截取部分数据展示，详见*_comp.combined.gtf）

seqname	source	feature	start	end	score	strand	frame	attributes
Chr01	StringTie	exon	69685	69903	.	+	.	gene_id "XLOC_000001"; transcript_id "TCONS_00000001"; exon_number "1"; gene_name "Glyma.01G000300"; oId "STRG.5.1"; nearest_ref "Glyma.01G000300.1"; class_code "c"; tss_id "TSS1";
Chr01	StringTie	exon	90211	90511	.	+	.	gene_id "XLOC_000002"; transcript_id "TCONS_00000002"; exon_number "1"; gene_name "Glyma.01G000500"; oId "STRG.1.1"; nearest_ref "Glyma.01G000500.1"; class_code "e"; tss_id "TSS2";
Chr01	StringTie	exon	90719	92779	.	+	.	gene_id "XLOC_000002"; transcript_id "TCONS_00000003"; exon_number "1"; gene_name "Glyma.01G000500"; oId "STRG.2.1"; nearest_ref "Glyma.01G000500.1"; class_code "e"; tss_id "TSS3";
Chr01	StringTie	exon	116094	118482	.	+	.	gene_id "XLOC_000003"; transcript_id "TCONS_00000004"; exon_number "1"; gene_name "Glyma.01G000600"; oId "STRG.4.1"; nearest_ref "Glyma.01G000600.2"; class_code "="; tss_id "TSS4";
Chr01	StringTie	exon	127392	127845	.	+	.	gene_id "XLOC_000003"; transcript_id "TCONS_00000004"; exon_number "2"; gene_name "Glyma.01G000600"; oId "STRG.4.1"; nearest_ref "Glyma.01G000600.2"; class_code "="; tss_id "TSS4";

(1) seqname：染色体编号
(2) source：来源标签，这里的novelGene指新基因
(3) feature：区域类型，目前我们预测外显子区域
(4) start：起始坐标
(5) end：终止坐标
(6) score：这个区域存在的可信值打分
(7) strand：正负链信息
(8) frame：如果是编码外显子，此处是0-2的数值；如果不是，就是“.”
(9) attributes：属性，包括基因编号、转录本编号等信息

Table 5.2 已知基因结构优化（截取部分数据展示，详见*_novel.xls）

Gene_id	Chromosome	Strand	Original_span	Assembled_span
Glyma.01G000300	Chr01	+	69600-69968	67770-69968
Glyma.01G000500	Chr01	+	90922-91197	90277-91197
Glyma.01G000600	Chr01	+	127392-127845	116094-127845
Glyma.01G000900	Chr01	+	192861-193342	143467-193364
Glyma.01G001000	Chr01	+	201524-201895	196256-201895

(1) Gene_id：基因命名编号
(2) Chromosome：染色体编号
(3) Strand：正负链信息
(4) Original_span：原注释文件中基因以及起始位置~终止位置
(5) Assembled_span：转录组拼接结果中基因起始位置~终止位置

6 SNV和InDel分析

All.xls
annotation.stat
*_Dist.png

6.1 SNV和InDel分析

我们通过各样本与参考基因组的比对结果，利用samtools（v0.1.18）软件进行mpileup处理，从而获取各样本可能的SNV结果，然后用annovar（v2013.02.11）软件分别进行注释。基于数据库中注释好的基因信息，该软件能够将突变信息与基因信息关联，从而实现对突变位点的注

释。突变引起氨基酸变化以及突变频率等信息请参考突变位点注释结果参见表6.1：

dbSNP数据库(version 135)是NCBI网站上专门用来收录已经报道过所有SNV和InDel信息（注：注释的结果需要在相同版本的数据库中查看）。

千人基因组(1000 genome)数据库(version 1000g2012apr)记录相关突变位点的突变频率信息。

Table 6.1.1 SNV分析结果（截取部分数据展示，详见All.xls）

Type	Chr	Start	END	Ref	Obs	Func	Gene	ExonicFunc	AAChange	1000genome	dbsnp	A35-2a/(hom/het)	Qual	D
SNV	scaffold_635	4537	4537	A	G	intergenic	NONE(dist=NONE),NONE(dist=NONE)			unknown	unknown	-	-	-
SNV	scaffold_635	4567	4567	T	G	intergenic	NONE(dist=NONE),NONE(dist=NONE)			unknown	unknown	-	-	-
SNV	scaffold_635	6157	6157	G	T	intergenic	NONE(dist=NONE),NONE(dist=NONE)			unknown	unknown	-	-	-
INDEL	scaffold_21	31863	31863	-	CAT	exonic	Glyma.U009200	unknown	UNKNOWN	unknown	unknown	-	-	-
INDEL	scaffold_21	175888	175888	-	TTC	upstream	Glyma.U010000			unknown	unknown	-	-	-

(1) Type:位点突变分类(SNV/InDel)
(2) Chr:染色体
(3) Start:起始位置
(4) End:终止位置
(5) Ref:参考碱基
(6) Obs:突变碱基
(7) Func:功能区位置分类
(8) Gene:功能区对应基因
(9) ExonicFunc:外显子功能区突变类型
(10) AAChange:碱基及氨基酸突变信息(NCBI序列号:碱基突变：氨基酸突变)
(11) 1000genome:1000genome突变频率
(12) dbsnp:SNP数据库注释ID
(13) Sample*:样本信息，分四列：
 ---hom/het 突变类型(heterozygous或homozygous)
 ---Qual ---质量
 ---Depth 碱基深度
 ---Freq 突变频率

AAChange示例说明：

Table 6.1.2

SNV示例	NM_177987:c.G729A:p.P243P
NM_177987	基因标示
c.	染色体
G	参考碱基
729	基因上的位置
A	突变碱基
p.	肽链
P	替换前的参考氨基酸
243	氨基酸在肽链上的位置
P	突变后氨基酸变化

Table 6.1.3

InDel示例	NM_014696:c.720_721insATGAGGGAG:p.E240delinsEMRE
NM_014696	基因标示
c.	染色体
720_721	基因位置720与721之间插入片段
ins insert	插入
p.	肽链
E240	肽链240位的氨基酸E后插入MRE这3个氨基酸
delins	插入
EMRE	插入后氨基酸变化情况

6.2 SNV/InDel在基因组功能区分布

通过数据库中基因注释信息，统计SNV/InDel相对于基因的发生位置即突变在基因组上的位置信息，并统计分布状况。

Table 6.2.1 SNV/InDel在基因组上的分布

Sample	A35-2a	A35-2b	A35-2c	A35-4a	A35-4b	A35-4c	A35-6a	A35-6b	A35-6c	A7-2a	A7-2b	A7-2c	A7-4a	A7-4b	A7-4c	A7-6a	A7-6b	A7-6c
exonic	37486	37259	38495	28336	34389	38271	33403	28556	21816	53684	50977	53920	46668	47340	44083	39882	39041	38159
intergenic	3392	4013	4649	2143	3007	4510	3493	2137	1351	7563	7114	8356	4566	4673	4322	2672	3323	2609
intronic	15033	16528	17529	8089	17001	23911	19423	11097	4290	29671	26720	31895	24005	26290	24029	10936	17252	11027
splicing	342	372	448	233	263	387	353	156	128	422	420	427	307	314	271	176	231	228
exonic;splicing	35	45	43	30	34	36	38	29	21	40	37	41	31	33	34	31	26	24
ncRNA_exonic	0	0	0	0	0	0	0	0	0	0	0	0	0	0	0	0	0	0
ncRNA_intronic	0	0	0	0	0	0	0	0	0	0	0	0	0	0	0	0	0	0
ncRNA_splicing	0	0	0	0	0	0	0	0	0	0	0	0	0	0	0	0	0	0
ncRNA_UTR3	0	0	0	0	0	0	0	0	0	0	0	0	0	0	0	0	0	0
ncRNA_UTR5	0	0	0	0	0	0	0	0	0	0	0	0	0	0	0	0	0	0
upstream	938	1043	1156	543	718	1022	819	516	357	1626	1440	1838	1033	1130	1017	624	757	653
downstream	1171	1492	1413	665	1108	1476	1181	874	429	2410	2254	2566	1391	1599	1338	835	1036	854
upstream;downstream	153	219	229	100	169	211	188	102	57	301	300	325	196	233	184	116	144	111
UTR3	0	0	0	0	0	0	0	0	0	0	0	0	0	0	0	0	0	0
UTR5	0	0	0	0	0	0	0	0	0	0	0	0	0	0	0	0	0	0
UTR5;UTR3	0	0	0	0	0	0	0	0	0	0	0	0	0	0	0	0	0	0
Total	58550	60971	63962	40139	56689	69824	58898	43467	28449	95717	89262	99368	78197	81612	75278	55272	61810	53665

(1) Sample: 测序样品名称
(2) exonic: 外显子区
(3) intergenic: 基因间区
(4) intronic: 内含子区
(5) splicing: 剪切位点
(6) exonic;splicing: 外显子区；剪切位点
(7) ncRNA_exonic: ncRNA外显子区
(8) ncRNA_intronic: ncRNA内含子区
(9) ncRNA_splicing: ncRNA剪切位点
(10) ncRNA_UTR3: ncRNA的3’ UTR区
(11) ncRNA_UTR5: ncRNA的5’ UTR区
(12) upstream: 基因上游区
(13) downstream: 基因下游区
(14) upstream;downstream: 基因上游区；其他基因下游区
(15) UTR3: 基因3’UTR区
(16) UTR5: 基因5’ UTR区
(17) UTR5;UTR3:基因5’ UTR区；其他基因3’ UTR区
(18) Total: 突变位点总数

通过饼图，可直观观测到各样本SNV/InDel在基因组不同功能区域分布状况，如下图所示：

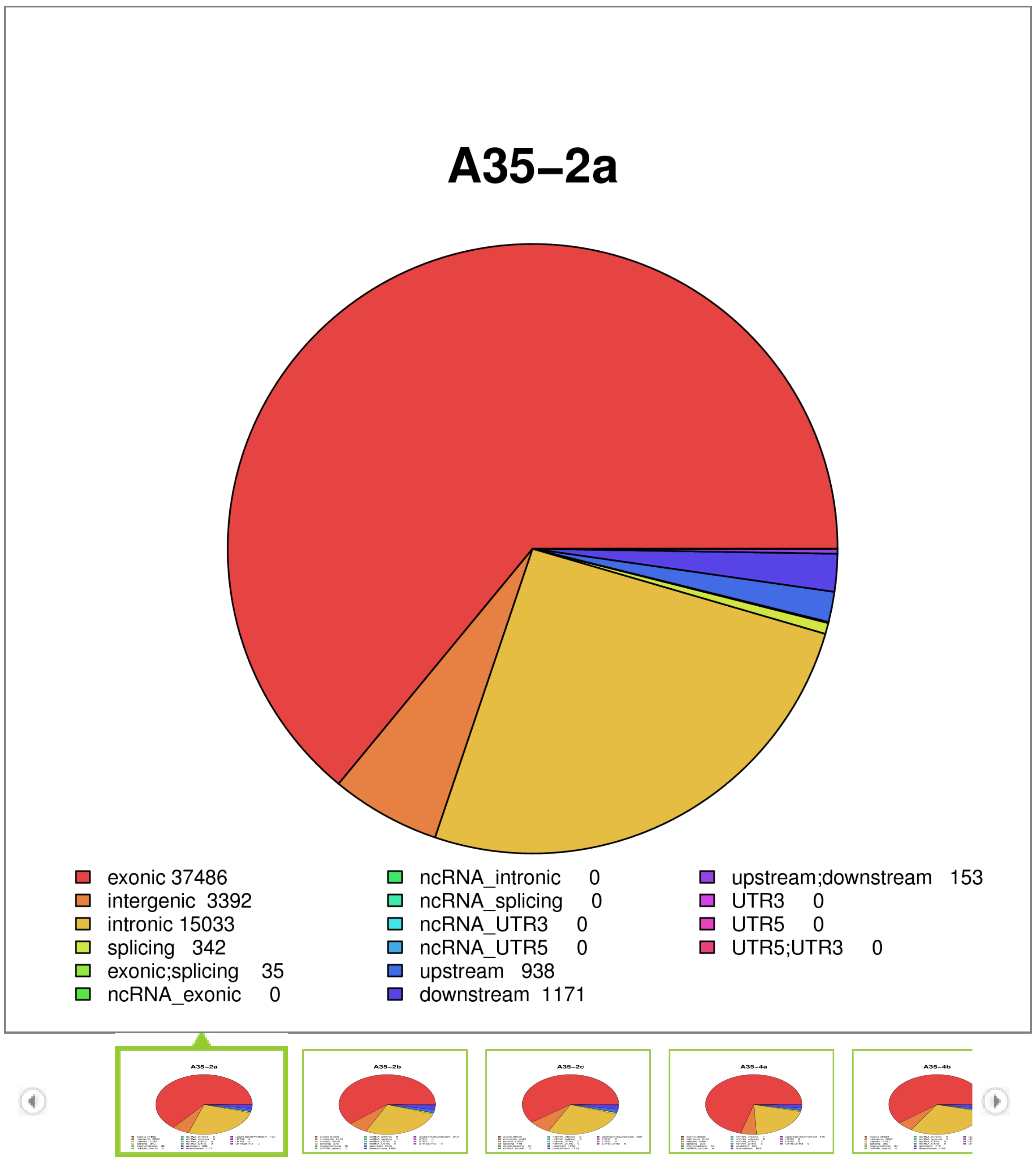

Figure 6.2.1 SNV/InDel在基因组上的分布

7 基因表达分析

RPKM_Region.log
all.rpkm.xls
*.png

一个基因表达水平的直接体现就是其在基因上的丰度情况，基因丰度程度越高，则基因表达水平越高。基因表达计算使用Htseq软件(V 0.6.1)，该软件使用RPKM（Reads Per Kilo bases per Million reads）（Mortazavi, 2008）方法计算基因表达量。

其公式为：

$$\text{RPKM} = \frac{total\ exon\ reads}{mapped\ reads\ (millions) * exon\ length\ (KB)}$$

Figure 7.1

其中，total exon reads / mapped reads (millions) 可以视为所有read 数中有百分之多少是map 到这个基因，然后再除以基因长度，就可以某基因得到单位长度有百分之多少的total mapped read 有表达。采用RPKM方法标准化不仅对测序深度作了归一化，而且对基因长度也作了归一化，使得不同长度的基因在不同测序深度下得到的基因表达水平估计值具有了可比性。

结果文件分别统计了不同表达水平下基因的数量以及单个基因的表达水平。一般情况下，RPKM 数值0.1 或者1 作为判断基因是否表达的阈值，不同的文献所采用的阈值不同。

Table 7.1 不同表达水平区间的基因数量统计表

Sample	0-0.1	0.1-1	1-3	3-15	15-60	>60
A35-2a	2458(5.63%)	8815(20.20%)	7959(18.24%)	16539(37.90%)	5996(13.74%)	1870(4.29%)
A35-2b	2224(5.15%)	8801(20.40%)	7883(18.27%)	16357(37.91%)	5948(13.78%)	1939(4.49%)
A35-2c	2363(5.54%)	8675(20.33%)	7842(18.38%)	16053(37.62%)	5786(13.56%)	1949(4.57%)
A35-4a	2602(6.64%)	9652(24.64%)	8605(21.97%)	13057(33.34%)	3927(10.03%)	1322(3.38%)
A35-4b	2524(6.18%)	8938(21.88%)	8389(20.53%)	15153(37.09%)	4422(10.82%)	1429(3.50%)
A35-4c	3388(8.17%)	8995(21.68%)	8007(19.30%)	15332(36.95%)	4493(10.83%)	1275(3.07%)
A35-6a	3105(7.94%)	9828(25.13%)	8644(22.10%)	13344(34.12%)	3278(8.38%)	908(2.32%)
A35-6b	2731(7.07%)	10074(26.08%)	8873(22.97%)	12849(33.27%)	3249(8.41%)	845(2.19%)
A35-6c	3316(9.25%)	11408(31.81%)	8779(24.48%)	9142(25.49%)	2439(6.80%)	781(2.18%)
A7-2a	3395(7.68%)	8605(19.46%)	6951(15.72%)	16796(37.98%)	6649(15.04%)	1823(4.12%)
A7-2b	2972(6.77%)	8709(19.84%)	7119(16.22%)	16678(37.99%)	6510(14.83%)	1908(4.35%)
A7-2c	3428(7.77%)	8723(19.77%)	6968(15.79%)	16599(37.61%)	6539(14.82%)	1873(4.24%)
A7-4a	3184(7.75%)	9089(22.13%)	8305(20.22%)	15126(36.83%)	4118(10.03%)	1248(3.04%)
A7-4b	2811(6.79%)	8618(20.83%)	7671(18.54%)	15905(38.44%)	4955(11.98%)	1417(3.42%)
A7-4c	2796(6.93%)	9246(22.93%)	8307(20.60%)	14939(37.05%)	3899(9.67%)	1134(2.81%)
A7-6a	3121(7.96%)	10421(26.58%)	9063(23.12%)	12669(32.32%)	3084(7.87%)	845(2.16%)
A7-6b	3023(7.84%)	10093(26.16%)	9033(23.41%)	12762(33.08%)	2918(7.56%)	754(1.95%)
A7-6c	3361(8.62%)	10253(26.31%)	8948(22.96%)	12618(32.38%)	3021(7.75%)	770(1.98%)

Table 7.2 基因表达水平统计表（截取部分数据展示，详见all.rpkm.xls）

gene_id	Exonic.gene.sizes	A35-2a	A35-2a_RPKM	A35-2b	A35-2b_RPKM	A35-2c	A35-2c_RPKM	A35-4a	A35-4a_RPKM	A35-4b	A35-4b_RPKM	A35-4c	A35-4c_RPKM	A35-6a	A35-6a_RPKM	A35-6b	A35-6b_R
Glyma.01G000100	718	97	8.49	76	7.49	39	3.22	78	7.25	139	13.09	266	15.04	153	10.81	82	8.76
Glyma.01G000200	1046	50	3.00	52	3.52	82	4.65	127	8.10	107	6.92	256	9.94	120	5.82	31	2.27
Glyma.01G000300	387	0	0.00	7	1.28	4	0.61	1	0.17	1	0.17	7	0.73	2	0.26	0	0.00
Glyma.01G000400	2459	244	6.24	179	5.15	220	5.31	122	3.31	200	5.50	343	5.66	178	3.67	79	2.46
Glyma.01G000500	357	6	1.06	1	0.20	10	1.66	0	0.00	5	0.95	16	1.82	2	0.28	2	0.43

8 RNA-seq整体质量评估

Cor
Saturation
Evenness

8.1 表达水平的饱和曲线检查

定量饱和曲线检查反映了基因表达水平定量对数据量的要求。表达量越高的基因，就越容易被准确定量；反之，表达量低的基因，需要较大的测序数据量才能被准确定量。软件RSeQC通过对总的比对reads重采样（jackknifing），评估在当前测序深度下的RPKMs,使用相对错误率Percent relative error来测量评估的RPKM的准确性。

$$\text{Percent Relative Error} = \frac{|RPKM_{obs} - RPKM_{real}|}{RPKM_{real}} \times 100$$

Figure 8.1.1

其中，$RPKM_{obs}$是指每一个百分比抽样下的当前转录本的RPKM值，$RPKM_{real}$是指总的数据量下当前转录本的RPKM值。

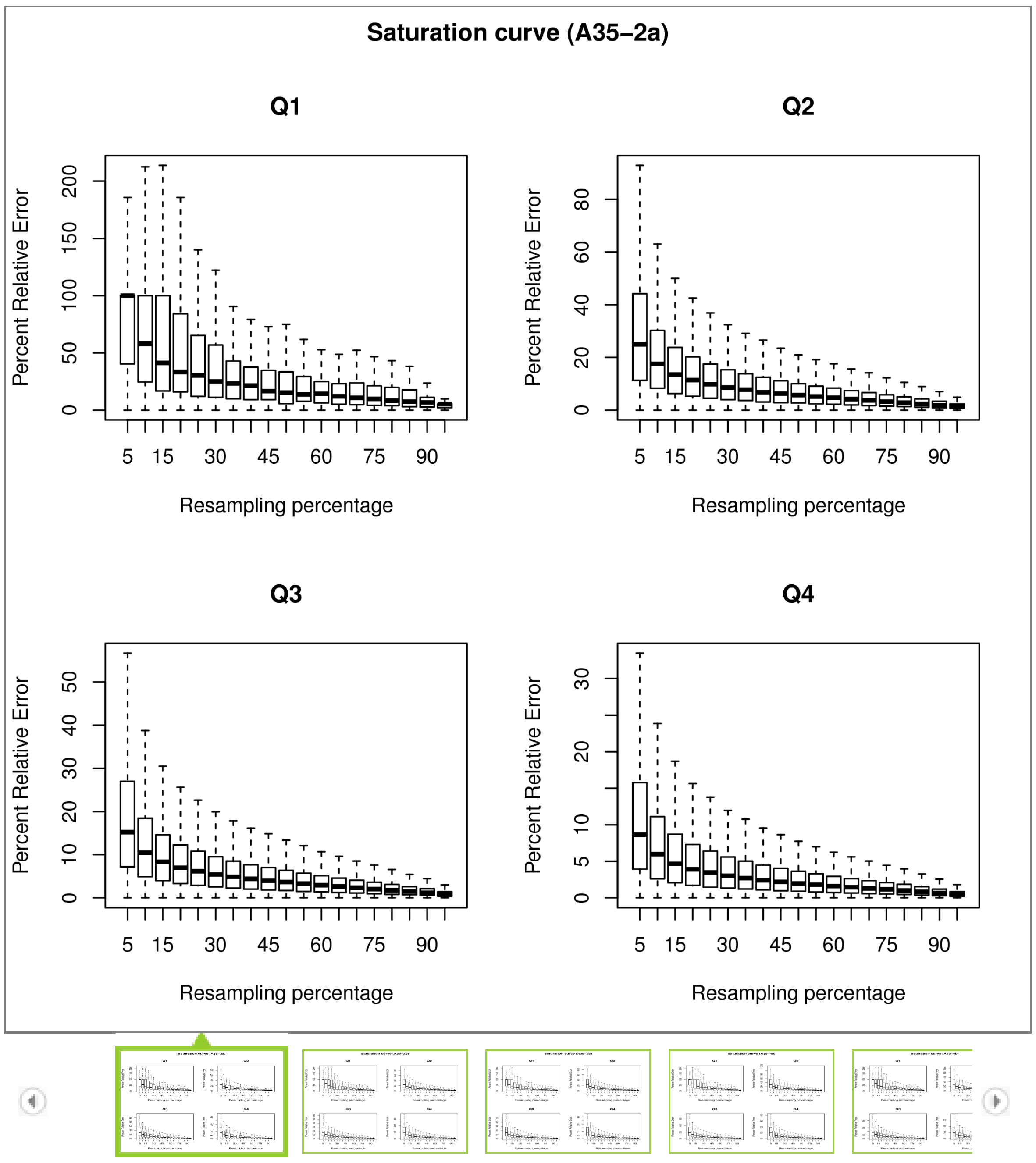

Figure 8.1.2 定量饱和曲线检查分布图，横坐标代表定位到基因组上的reads数占总reads数的百分比，纵坐标代表相对误差百分比。Q1为转录本表达水平低于25%的饱和度箱图；Q2为转录本表达水平在25%~50%的饱和度箱图；Q3为转录本表达水平在50%~75%的饱和度箱图；Q4为转录本表达水平高于75%的饱和度箱图。

8.2 RNA-Seq相关性检查

生物学重复主要有两个用途：一个是证明所涉及的生物学实验操作是可以重复的且变异不大，另一个为后续的差异基因分析所需要的。样品间基因表达水平相关性是检验实验可靠性和样本选择是否合理性的重要指标。Pearson correlation Coefficient是一个相关系数，它指出了两个变量之间相关的亲密程度和方向。这个数值的绝对值越大越说明两个变量的关系越亲密，它的绝对值为0-1之间。具体的项目操作中，我们要求R2至少要大于0.8，否则需要对样品做出合适的解释，或者重新进行实验。此部分，我们同时计算了spearman 秩相关系数和kendall-tau等级相关系数作为参考。

如果没有生物学重复，则不进行该项分析，即结果文件夹Cor为空。

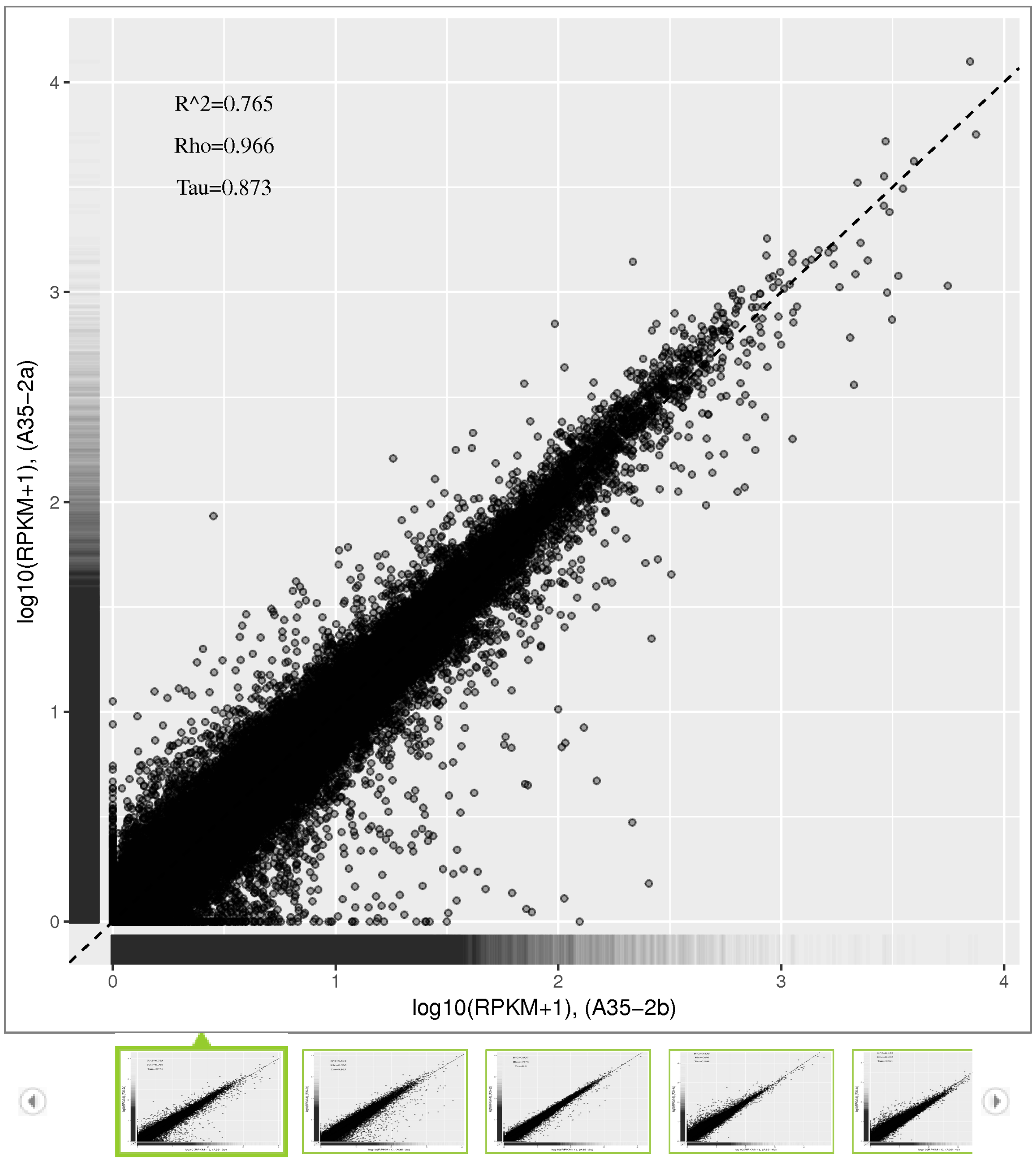

Figure 8.2.1 RNA-Seq相关性检查

R^2:pearson 相关系数的平方; rho:spearman 相关系数; tau:kendall-tau 相关系数。

8.3 均一性分布检查

理想条件下，对于RNA-seq 技术来说，测序reads之间为独立抽样并且reads 在所有表达的转录本上的分布应该呈现均一化分布。然而很多研究表明，很多偏好型的因素都会影响这种均一化的分布(Dohm et al., 2008)。例如，在RNA-seq 建库过程中，片段破碎和RNA 反转录的顺序不一样会导致RNA-seq 最终的数据呈现严重的3’偏好性。其他因素还包括转录区域的GC含量不同、随机引物等等，并且生物体内从5’或者3’的降解过程同样会导致不均一性分布。

均一性分布的曲线算法是：

(1) 把每个转录本从5’到3’的方向平均等分为100份
(2) 计算每个等份中的碱基平均测序深度，并用最大值来进行归一化处理

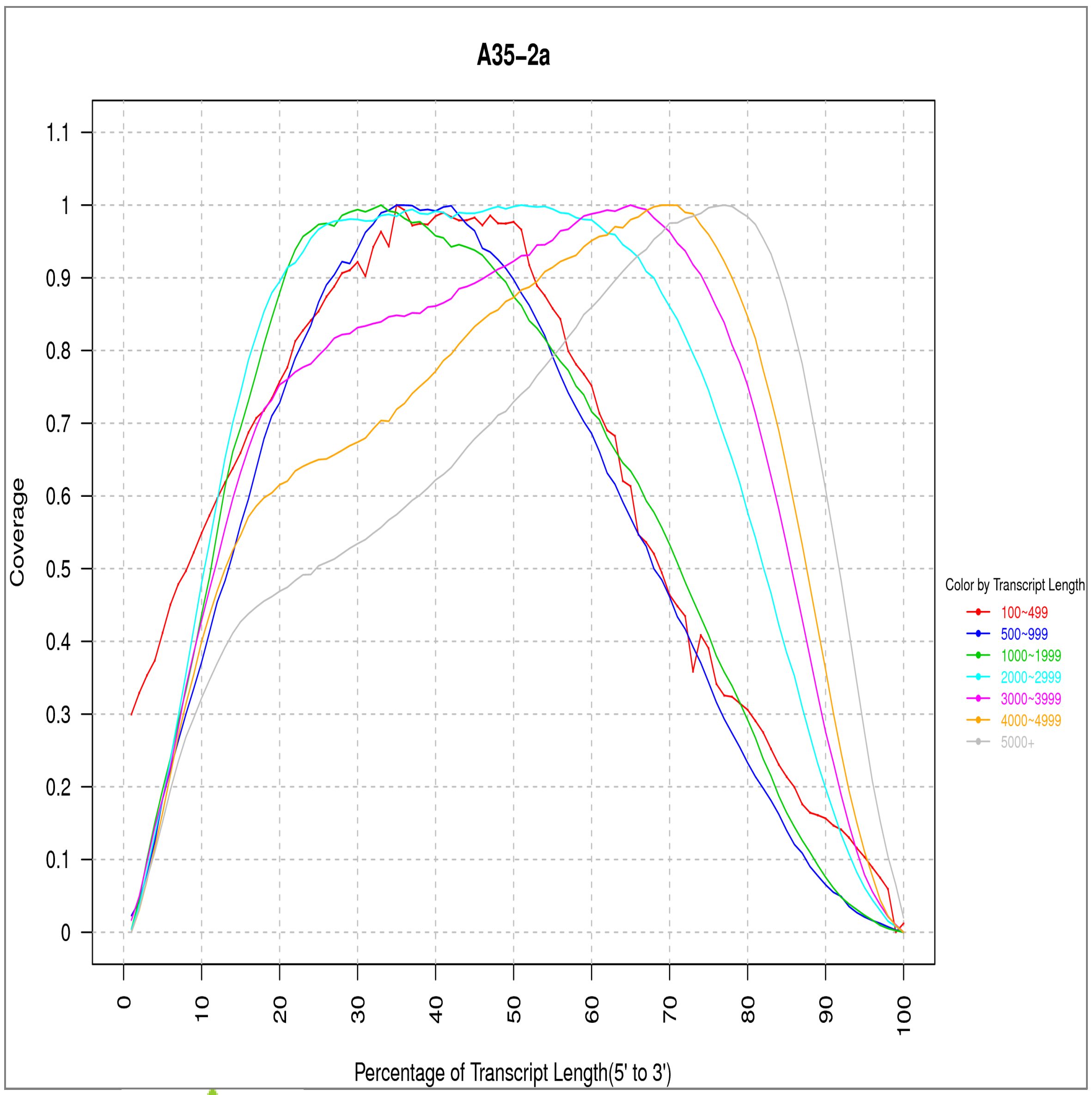

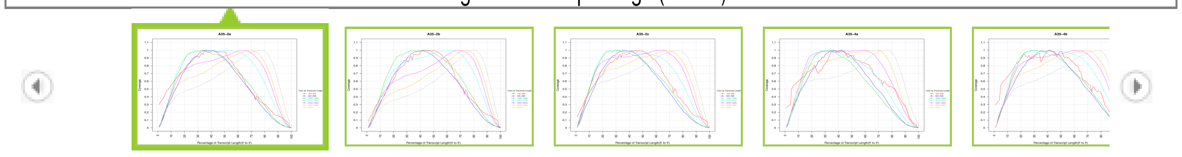

Figure 8.3.1 不同长度的转录本的reads 密度分布图，横坐标为转录本长度的百分比；纵坐标是平均测序深度；不同的颜色表示不同长度范围的转录本

9 PCA分析

PCA.png

PCA(Principal Component Analysis),即主成分分析可以降低数据的复杂性，深入挖掘样品之间关系和变异大小。基本原理是利用数学的方法，将原来变量重新组合成一组新的互相无关的几个综合变量（即主成分），对所有因素按重要性排序，通常靠后的微小因素被忽略，从而起到简化数据的作用。通常以两个或三个主成分为坐标轴画成图，就可以看出各个样本之间的距离关系，包括成簇成组的视觉效果。下图展示样本间的聚类关系：

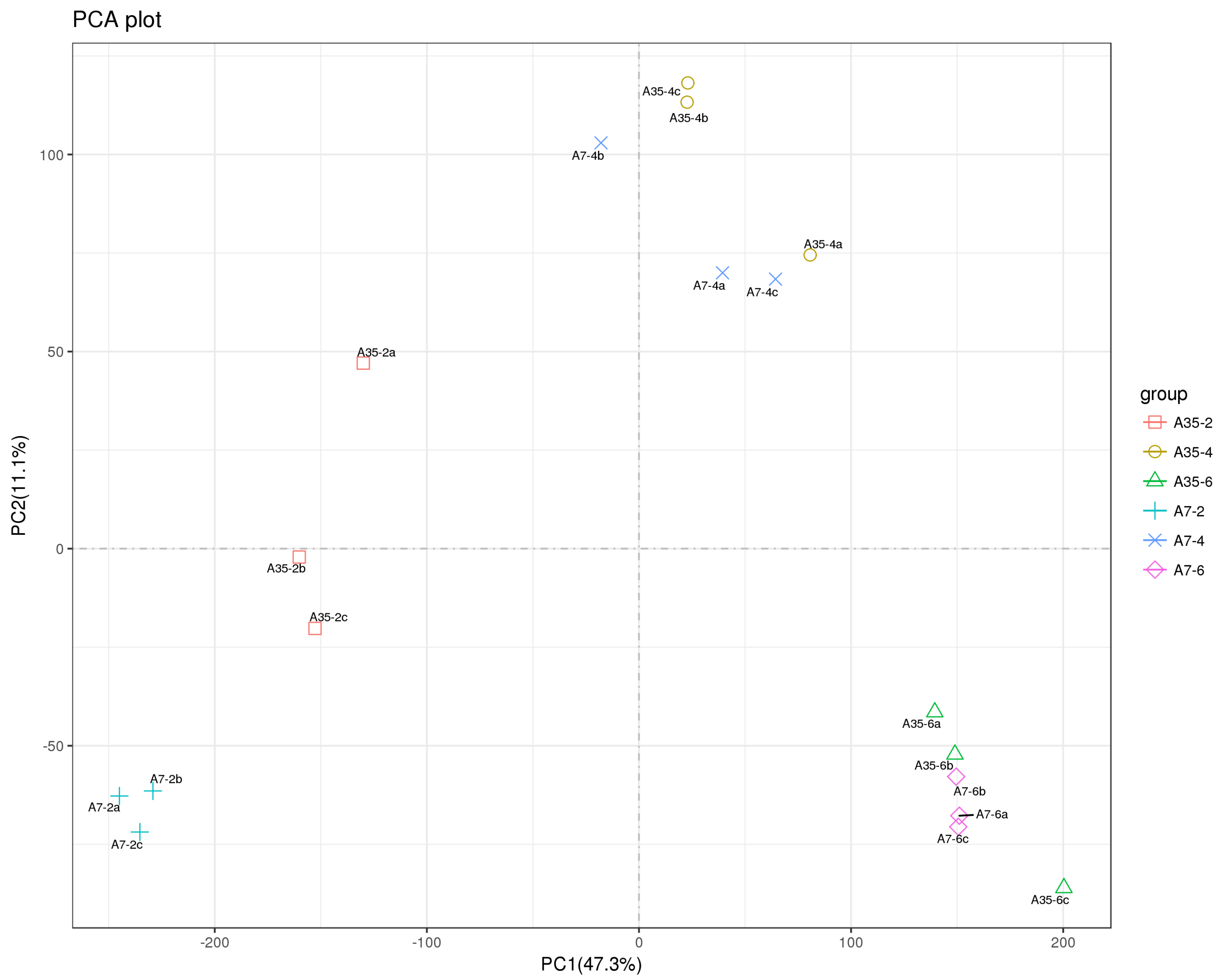

Figure 9.1 主成分分析图，每个圆点的位置代表样品在各主成分上的取值

10 基因差异表达分析

- *_DE_anno.xls
- GroupDE_significant_UpDown*
- *MA_Volcano-1.png
- diffExpr.P0.05_C1.matrix.heatmap-0.png
- venn.png

10.1 基因表达水平对比

通过所有基因的 RPKM 的分布图以及盒形图对不同实验条件下的基因表达水平进行比较。

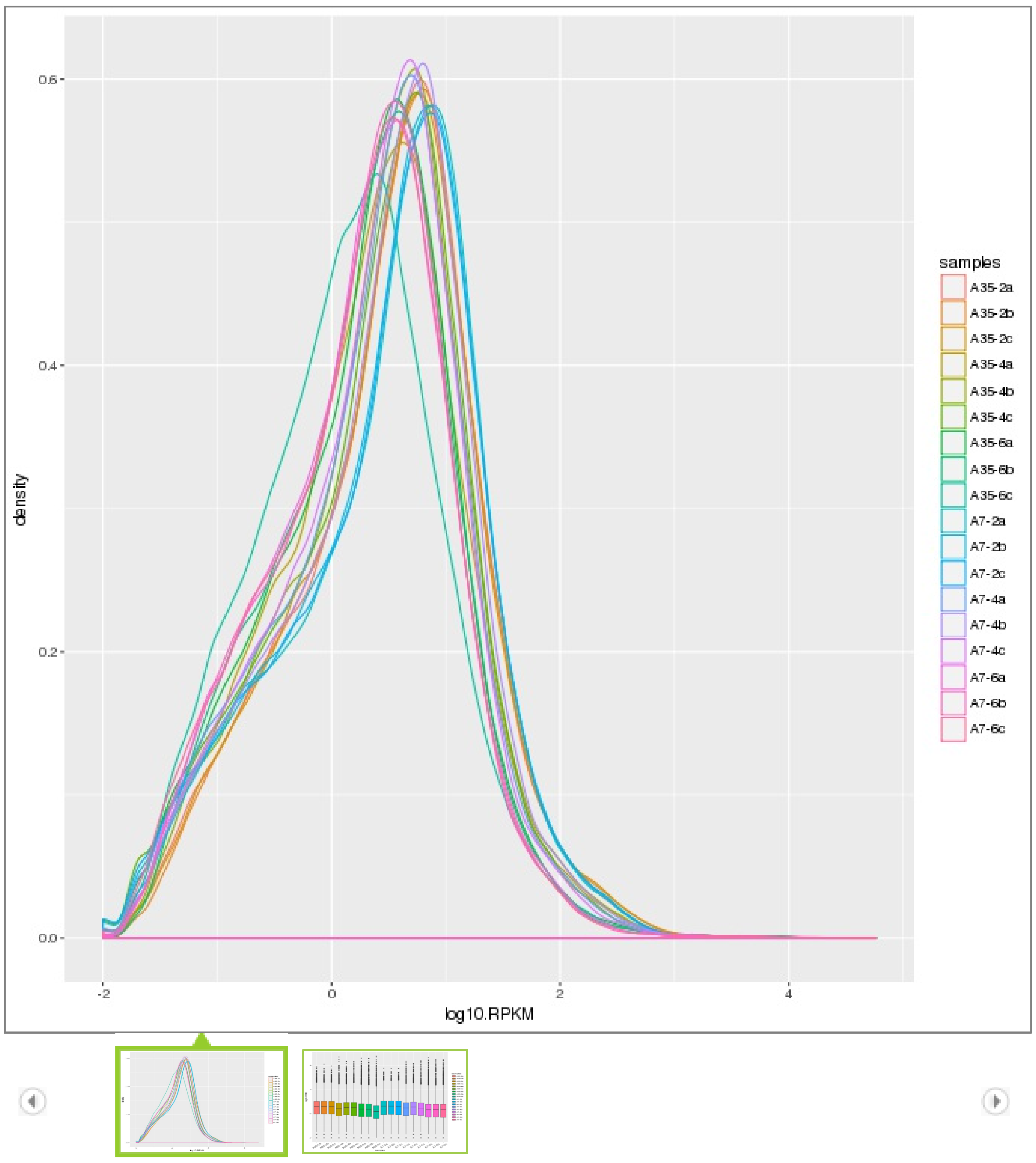

Figure 10.1.1 不同实验条件下基因表达水平比对图 图一：RPKM分布图，横坐标为log_{10}(RPKM), 纵坐标为基因的密度。图二：RPKM盒形图，横坐标为样品名称，纵坐标为log_{10}(RPKM)，每个区域的盒形图对五个统计量(至上而下分别为最大值，上四分位数，中值，下四分位数和最小值)

10.2 差异表达基因列表

基因差异表达的输入数据为基因表达水平分析中得到的read count数据。

对于有生物学重复的样品，基因差异分析使用Bioconductor软件包的DESeq2（V1.6.3）进行分析,该分析方法基于的模型是负二项分布，第 i 个基因在第 j 个样本中的 read count 值为K_{ij}，则有

$$K_{ij} \sim NB(\mu_{ij},\alpha_{ij})$$

$$\mu_{ij}=s_j q_{ij}$$

$$log_2=x_j\beta_i$$

特殊情况下，基因差异分析使用Bioconductor软件包的edgeR（V3.4.6）进行分析。

本次分析使用的软件是DESeq2。

Table 10.2.1 差异基因列表（截取部分数据展示，详见*_DE.xls）

gene_id	baseMean	log2FoldChange	lfcSE	stat	pvalue	padj	GeneSymbol
Glyma.11G065800	4774.04026438279	9.15450057068815	0.240392862747382	38.0814158376582	0	0	Glyma.11G06580[illegible]
Glyma.11G252800	11573.2117931042	11.056708962063	0.270194420913998	40.9213074224886	0	0	Glyma.11G25280[illegible]
Glyma.U020300	10198.212047409	9.25872585530302	0.246008973205302	37.63572415538	0	0	Glyma.U020300
Glyma.08G350800	8591.11038630432	5.32045961890756	0.14189616808124	37.4954425538921	1.09285837160669e-307	1.16821095632897e-303	Glyma.08G35080[illegible]
Glyma.20G219900	1556.67124947904	4.21581619802145	0.115300650084312	36.5636810801906	1.08097855810635e-292	9.24409623750223e-289	Glyma.20G21990[illegible]

(1) Id:基因ID
(2) baseMean:片段数目的平均值归一化，两种条件下的平均值
(3) log2FoldChange:对差异变化值以2为底取log
(4) lfcSE:对log2FoldChange进行标准误差估算
(5) stat:Wald检验
(6) pval:统计差异显著性值
(7) padj:FDR校正P值
(8) GeneSymbol:基因名称
(9) KO:KEGG ID
(10) GO:Gene Ontology ID

10.3 差异表达基因筛选

对检测的结果按照差异显著性标准（差异基因表达变化2倍以上且FDR<=0.05）进行筛选，统计基因显著性差异表达上下调情况。

Table 10.3.1 不同条件样品基因显著性差异表达上下调数量

Sample-VS-Sample	UPs	Down
A7-2-VS-A7-4	5401	7237
A7-2-VS-A7-6	7299	9736
A7-4-VS-A7-6	3399	4196
A35-2-VS-A35-4	3230	6134
A35-2-VS-A35-6	5403	8828
A35-4-VS-A35-6	3341	4034
A35-2-VS-A7-2	1501	3317
A35-4-VS-A7-4	662	709
A35-6-VS-A7-6	822	693
A35-VS-A7	587	725

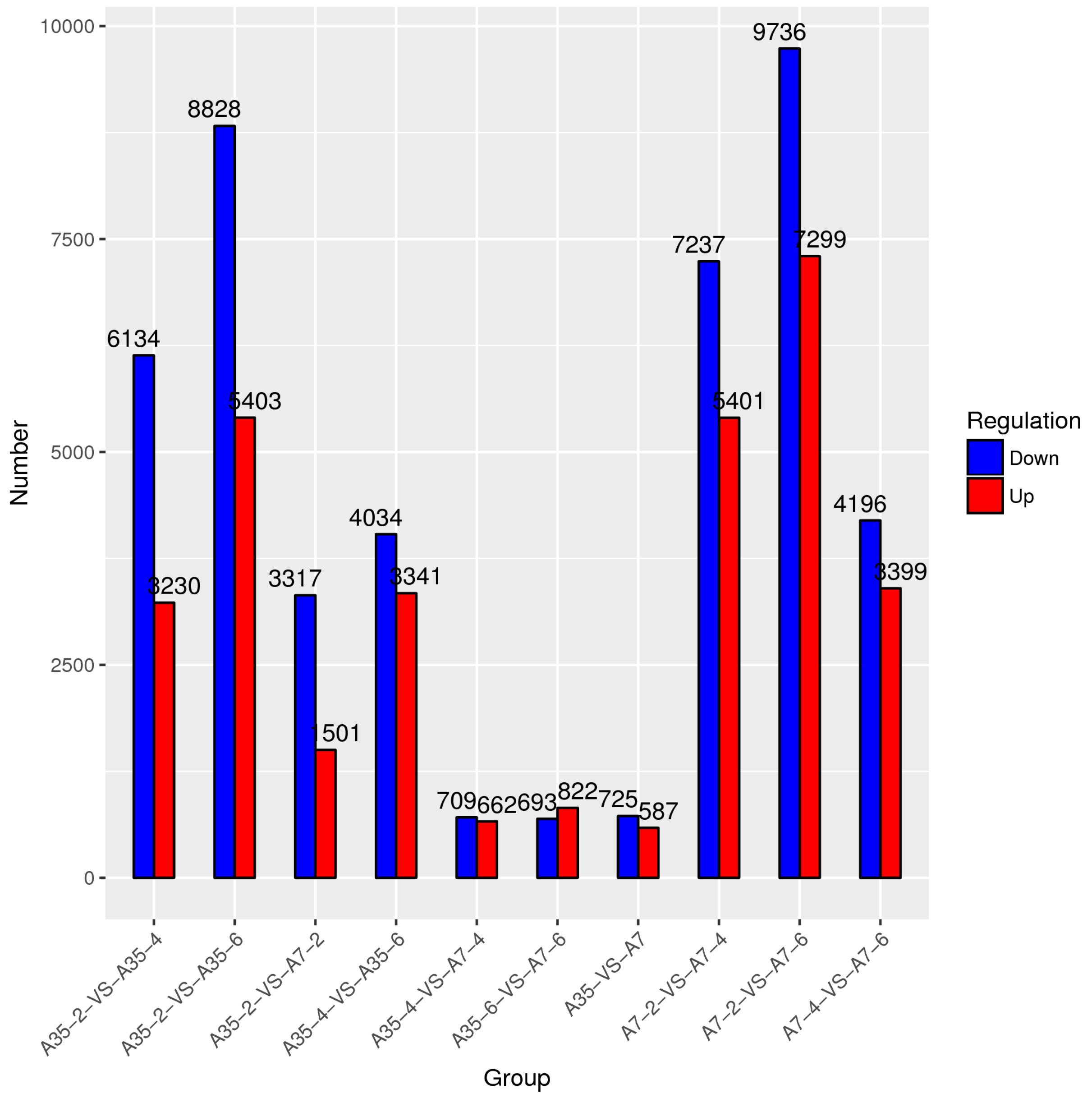

Figure 10.3.1 样品差异比较基因表达的上下调情况

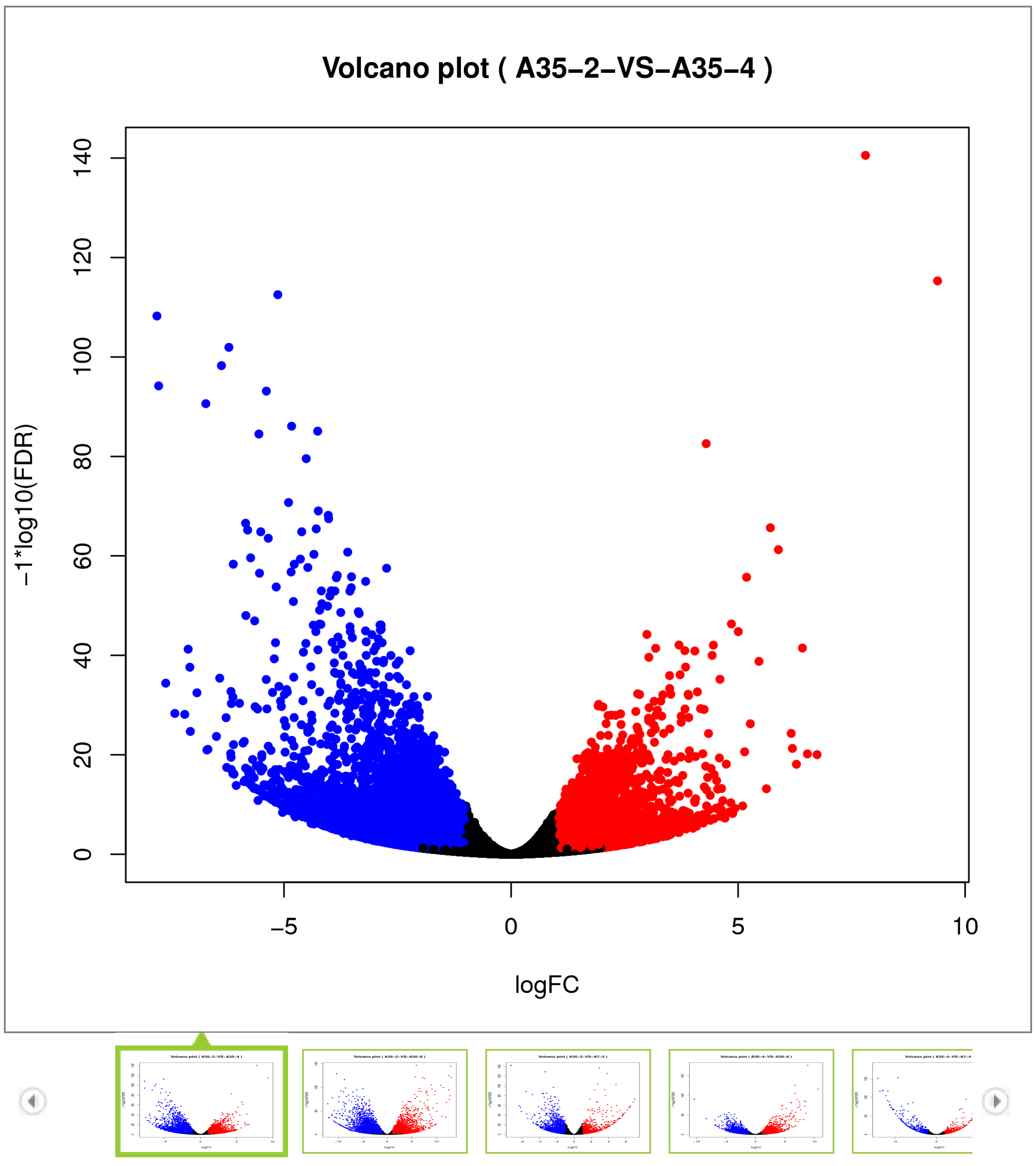

Figure 10.3.2 差异基因火山图，显著差异基因红色点表示上调，蓝色点表示下调；横坐标代表基因在不同样本中表达倍数变化；纵坐标代表基因表达量变化差异的统计学显著性

10.4 差异表达基因聚类分析

聚类分析是对数据进行相似度计算，并根据相似度将数据进行分类，从而将具有相同功能或密切联系的基因聚集成类，识别未知基因的功能或已知基因的未知功能，推断是否共同参与同一代谢过程或细胞通路。以不同实验条件下的差异基因的RPKM 值为表达水平，做层次聚类(hierarchical clustering)分析，这个方法最大的特征就是易于生成树状图。不同的颜色的区域代表不同的聚类分组信息，同组内的基因表达模式相近，可能具有相似的功能或参与相同的生物学过程。

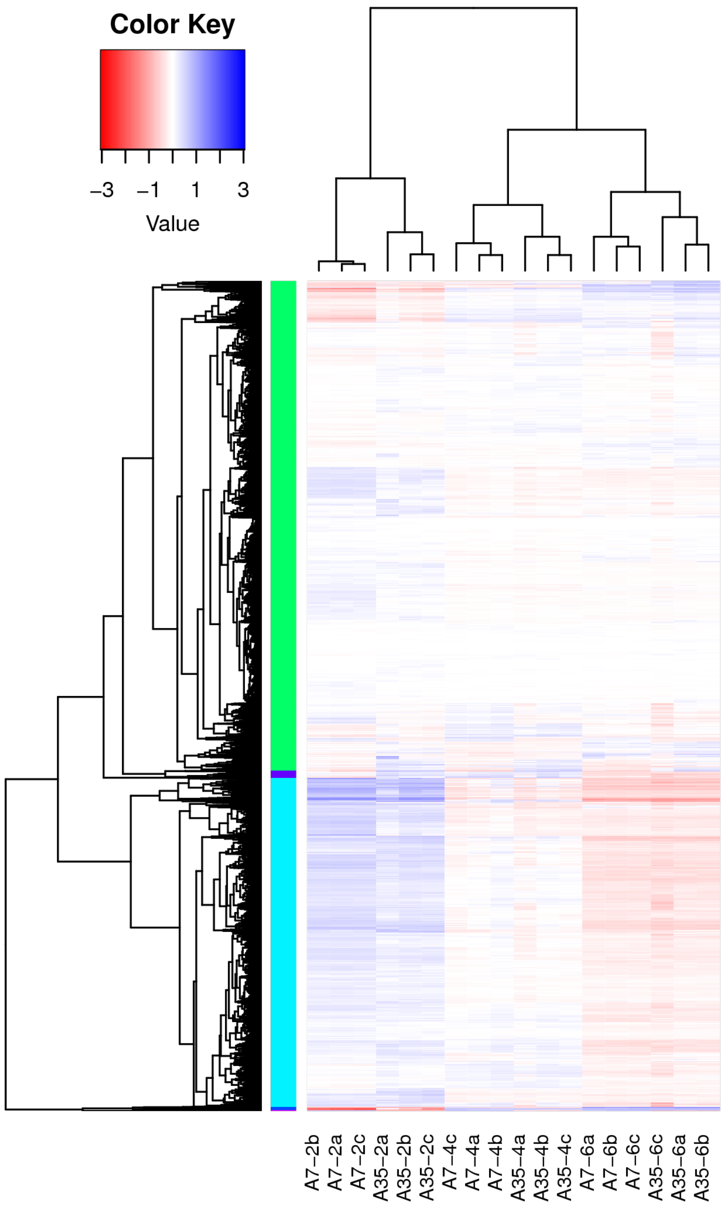

Figure 10.4.1 差异基因聚类图
以log10（RPKM+1） 值进行聚类，蓝色表示高表达基因，红色表示低表达基因。颜色从红到蓝，表示基因表达量越高。

10.5 差异基因维恩图

具体分析过程中，如果gene id一致时，则判定为差异基因为一致的。维恩图展示了样品两两间特有差异基因的数量，以及共有差异基因的数量(组数2-5，超出该范围则不作Venn图)。

11 差异基因GO富集分析

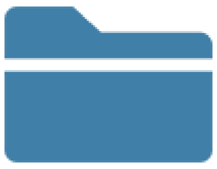

*.html
*.GoEnrichBar.png
*_P/C/F.png

Gene Ontology（GO）是一个国际标准化的基因功能分类体系，提供了一套动态更新的标准词汇表（controlled vocabulary）来全面描述生物体中基因和基因产物的属性。GO包含三个ontology，分别描述基因的分子功能（molecular function）、细胞组分（cellular component）、参与的生物过程（biological process）。

GO功能显著性富集分析给出与基因组背景相比，在差异表达基因中显著富集的GO功能条目，从而给出差异表达基因与哪些生物学功能显著相关。该分析首先把所有差异表达基因向Gene Ontology数据库的各个term映射，计算每个term的基因数目，然后应用超几何检验，找出与整个基因组背景相比，在差异表达基因中显著富集的GO条目，其计算公式为：

$$P = 1 - \sum_{i=0}^{m-1} \frac{\binom{M}{i}\binom{N-M}{n-i}}{\binom{N}{n}}$$

Figure 11.1

其中，N为所有基因中具有GO注释的基因数目；n为N中差异表达基因的数目；M为所有基因中注释为某特定GO term的基因数目；m为注释为某特定GO term的差异表达基因数目。计算得到的p-value通过Bonferroni校正之后，以corrected p-value≤0.05为阈值，满足此条件的GO term定义为在差异表达基因中显著富集的GO term。通过GO功能显著性富集分析能确定差异表达基因行使的主要生物学功能。

11.1 差异基因GO富集列表

Table 11.1.1 差异基因GO富集结果示例（详见：batchGOView.html）

Gene Ontology term	Cluster frequency	Genome frequency of use	Corrected P-value	FDR	False Positive	Genes annotated to the term
catalytic activity	13 out of 23 genes, 56.5%	914 out of 3874 genes, 23.6%	8.22e-05	0.23	0	gene1,gene2...

(1) Gene Ontology term：GO的类别
(2) Cluster frequency：聚类频率, 即目标基因中注释到该类别的基因比例
(3) Genome frequency of use：GO数据库中，注释到该类别的基因占所有基因的比例
(4) Corrected P-value：校正的P-value
(5) FDR：错误发现率
(6) False Positive：假阳性
(7) Genes annotated to the term：注释到此类别的基因列表

11.2 差异基因GO富集DAG图

有向无环图(Directed Acyclic Graph，DAG)为差异基因GO富集分析结果的图形化展示方式，分支代表包含关系，从上至下所定义的功能范围越来越小，一般选取GO富集分析的结果前10位作为有向无环图的主节点，并通过包含关系，将相关联的GO Term一起展示，颜色的深浅代表富集程度。分别对生物过程(biological process)、分子功能(molecular function)和细胞组分(cellular component)绘制DAG图。

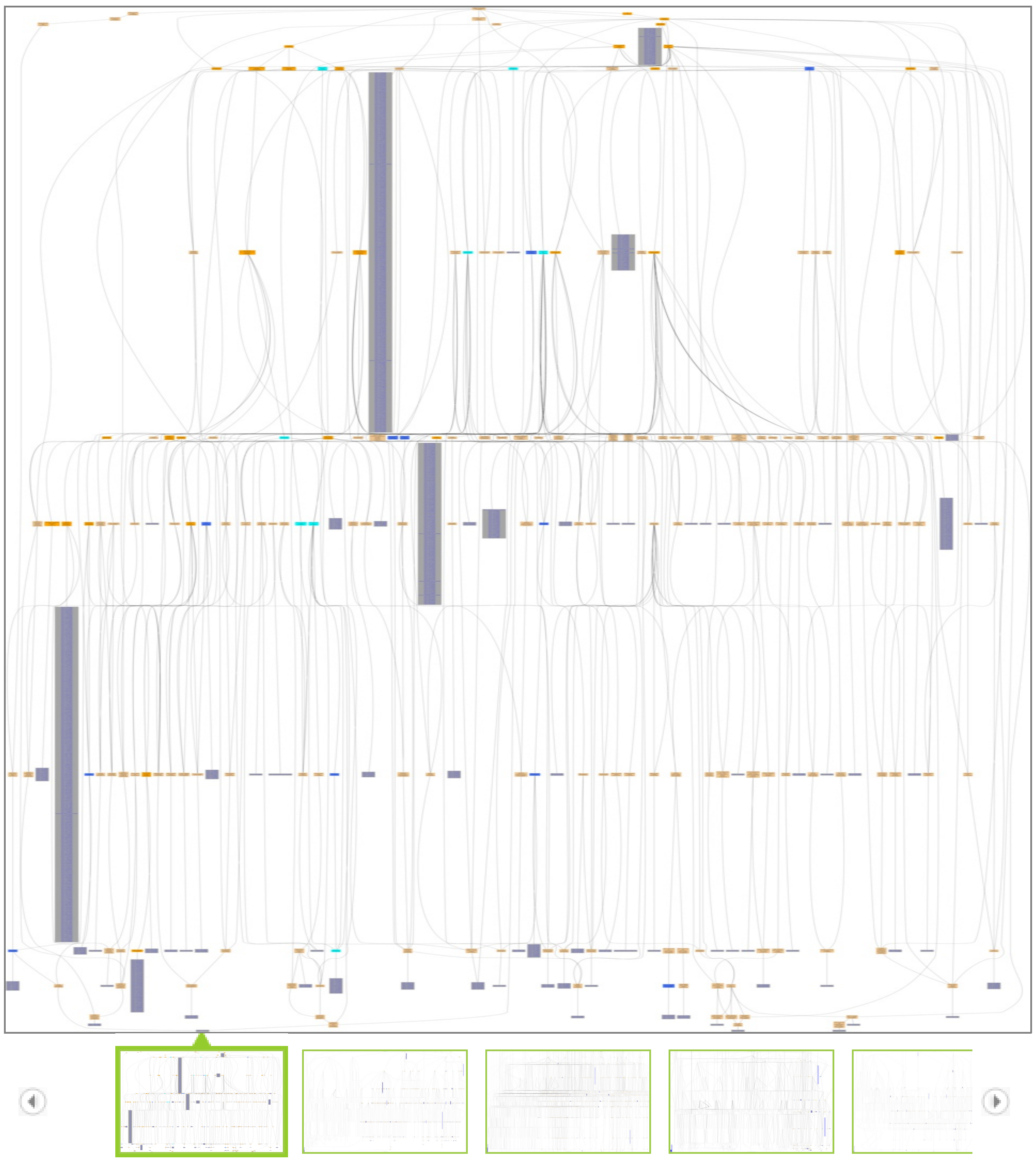

Figure 11.2.1 GO富集有向无环图

图中蓝色字体标所在GO term层级下的基因列表，GO term的颜色代表富集程度，由Corrected P-value 来标定。

P-value	颜色
<=1e-10	
1e-10 to le-8	
le-8 to le-6	
le-6 to le-4	
le-4 to le-2	
>0.01	

Figure 11.2.2

11.3 差异基因GO富集柱状图

差异基因GO富集柱状图，直观的反映出在生物过程(biological process)、细胞组分(cellular component)和分子功能(molecular function)富集的GO term上差异基因的个数分布情况。我们挑选了富集最显著的30个GO term在图中展示，如果不足30条，则全部展示。

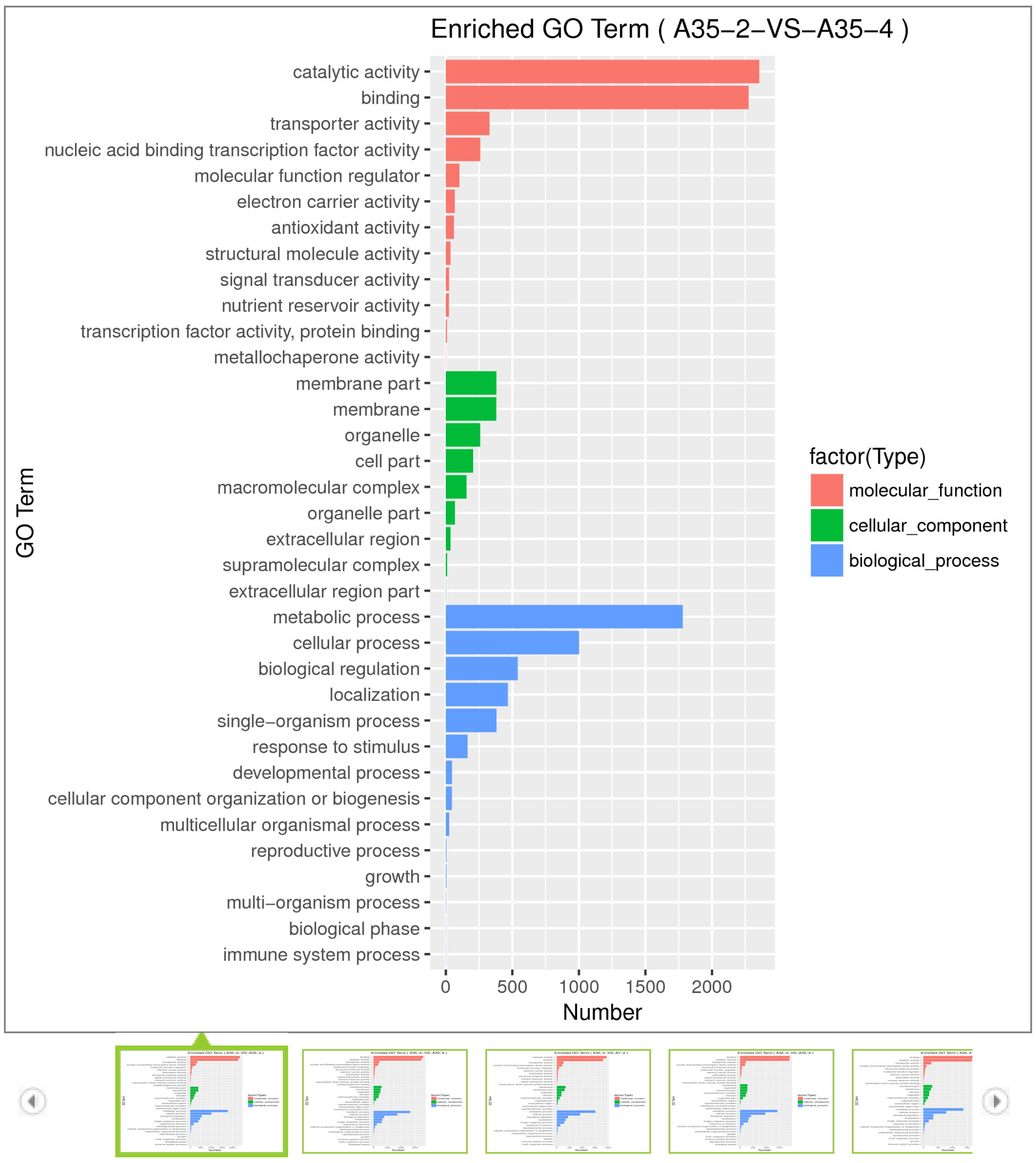

Figure 11.3.1 GO富集柱状图，纵坐标为富集的GO term，横坐标为该term中差异基因个数。不同颜色用来区分生物过程、细胞组分和分子功能。

12 差异基因KEGG富集分析

*_DE_significant_path.xls
Rich/
Pathway/

在生物体内，不同基因相互协调行使其生物学功能，通过Pathway显著性富集能确定差异表达基因参与的最主要生化代谢途径和信号转导途径。KEGG（Kyoto Encyclopedia of Genes and Genomes）是有关Pathway的主要公共数据库(Kanehisa,2008）。Pathway显著性富集分析以

KEGG Pathway为单位，应用超几何检验，找出与整个基因组背景相比，在差异表达基因中显著性富集的Pathway。该分析的计算公式:

$$p = 1 - \sum_{i=0}^{m-1} \frac{\binom{M}{i}\binom{N-M}{n-i}}{\binom{N}{n}}$$

Figure 12.1

在这里N为所有基因中具有Pathway注释的基因数目； n为N中差异表达基因的数目；M为所有基因中注释为某特定Pathway的基因数目； m 为注释为某特定Pathway的差异表达基因数目。

12.1 差异基因KEGG富集列表

Table 12.1.1 差异基因KEGG富集列表 示例（详见：*DE_significant_path.xls）

PathwayID	Pathway	DEGs with pathway annotation	All genes with pathway annotation	Pvalue	Qvalue	Genelist	KOlist
ko00982	Drug metabolism - cytochrome P450	1(7.69%)	7(0.42%)	1.15e-03	1.15e-03	gene1,gene2...	ko1,ko2..

(1) PathwayID:通路ID
(2) Pathway:通路名称
(3) DEGs with pathway annotation:该通路在差异分析比较组中基因数量
(4) All genes with pathway annotation :该通路在所有基因背景中的基因数量
(5) Pvalue:该通路富集Pvalue值
(6) Qvalue:该通路富集Qvalue值
(7) Genelist:该通路富集差异基因列表
(8) KOlist:该通路富集差异基因KO列表

12.2 差异基因KEGG富集散点图

散点图是 KEGG 富集分析结果的图形化展示方式。在此图中，KEGG 富集程度通过Rich factor、Qvalue 和富集到此通路上的基因个数来衡量。其中Richfactor 指差异表达的基因中位于该pathway 条目的基因数目与所有有注释基因中位于该pathway 条目的基因总数的比值。Rich factor 越大，表示富集的程度越大。Qvalue 是做过多重假设检验校正之后的Pvalue，Qvalue 的取值范围为[0,1]，越接近于零，表示富集越显著。我们挑选了富集最显著的30 条pathway 条目在该图中进行展示，若富集的pathway 条目不足30 条，则全部展示。

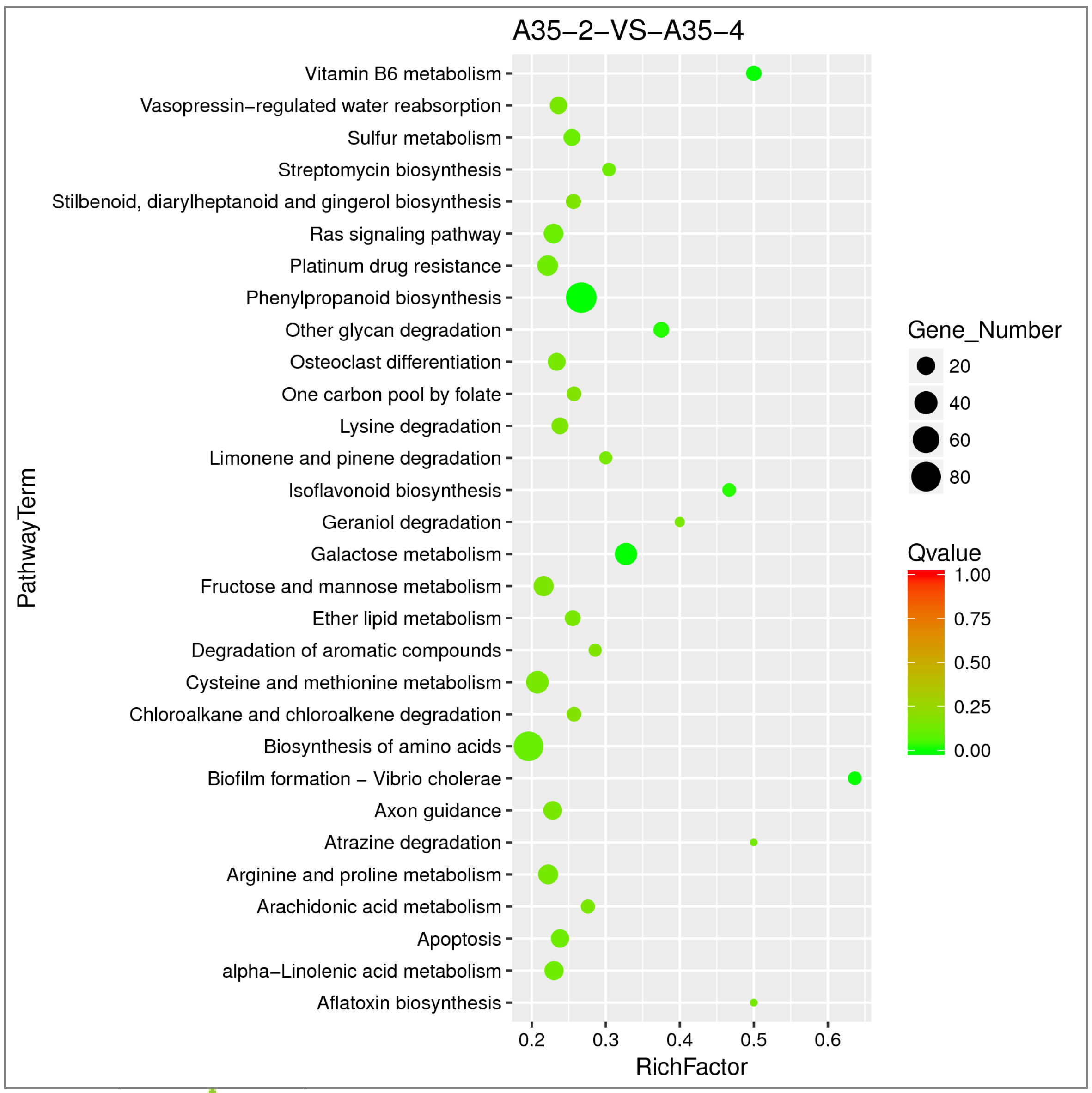

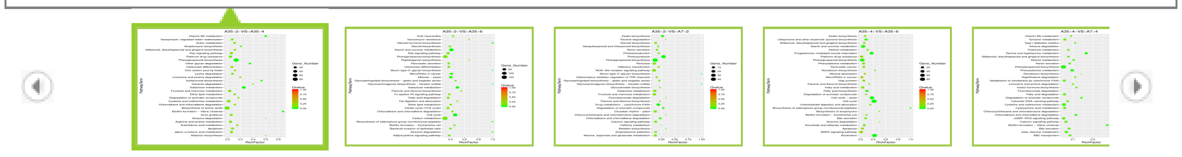

Figure 12.2.1 差异基因KEGG富集散点图，纵轴表示pathway名称，横轴表示Rich factor，点的大小表示此pathway中差异表达基因个数多少，而点的颜色对应于不同的Qvalue范围

12.3 富集KEGG通路图

将差异基因富集出的通路图展示出来，该通路图中，红色代表基因表达上调，蓝色代表基因表达下调，绿色代表基因既有上调也有下调。

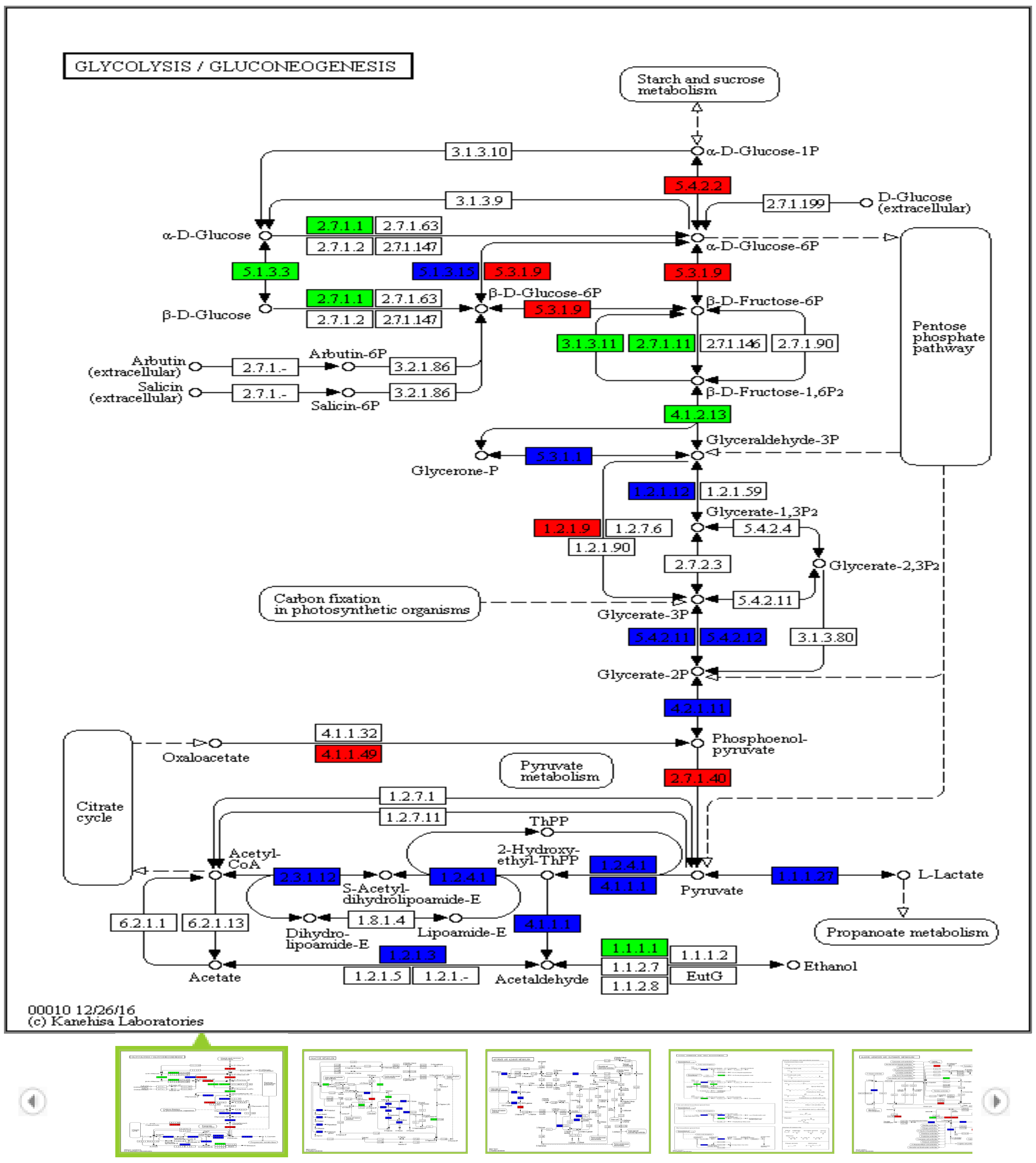

Figure 12.3.1 显著富集的KEGG pathway代谢通路图

13 DEU分析

DEXSeqResultTable0.05.csv
*.png

对于RNA-seq，除了gene水平的差异分析外，还可以进行DEU(differential exon usage)，即外显子水平的差异分析。DEU分析是目前最好的用于研究可变剪切中AEU(alternative exon usage)的方法。使用DEXSeq（V1.18.4）软件进行DEU分析。DEXSeq使用广义线性模型，可以在外显子水平上检测基因的差异表达。对DEU基因筛选的标准为：padjust < 0.05。DEU基因各外显子表达情况详细信通过如下图表展示，图中高亮紫红色block代表表达水平差异显著的外显子。

此分析只针对于有生物学重复的样本。如果没有生物学重复，则不进行此项分析。

Table 13.1 DEU基因列表

groupID	GeneID	featureID	exonBaseMean	dispersion	stat	pvalue	padj	A
Glyma.06G267400:E007	Glyma.06G267400	E007	1.48237148354453	0.0331191867255544	25.5233882681827	4.37052672714306e-07	0.0092930509799243	1
Glyma.13G279600:E002	Glyma.13G279600	E002	1.19766475486415	0.0229274008671676	26.4042503054059	2.76930436928549e-07	0.0092930509799243	2
Glyma.13G279600:E001	Glyma.13G279600	E001	1.42400769867562	0.0516748622078059	24.1007700712686	9.1423729162232e-07	0.0129596183545103	2

(1) groupID：基因编号和外显子编号
(2) GeneID：基因编号
(3) featureID：外显子编号
(4) exonbasemean：矫正后的平均表达量

(5) dispersion：统计学值，离差
(6) pvalue：统计学显著水平
(7) padjust：矫正后的统计学显著水平
(8) ctrl：ctrl组的表达值
(9) expr：expr组的表达值
(10) log2fold_ctrl_expr：矫正的差异表达倍数
(11) genomicData.seqnames ：染色体编号
(12) genomicData.start ：基因的起始位点
(13) genomicData.end ：基因的终止位点
(14) countData.Sample: 各样本的counts数
(15) transcripts: 基因上面的转录本编号

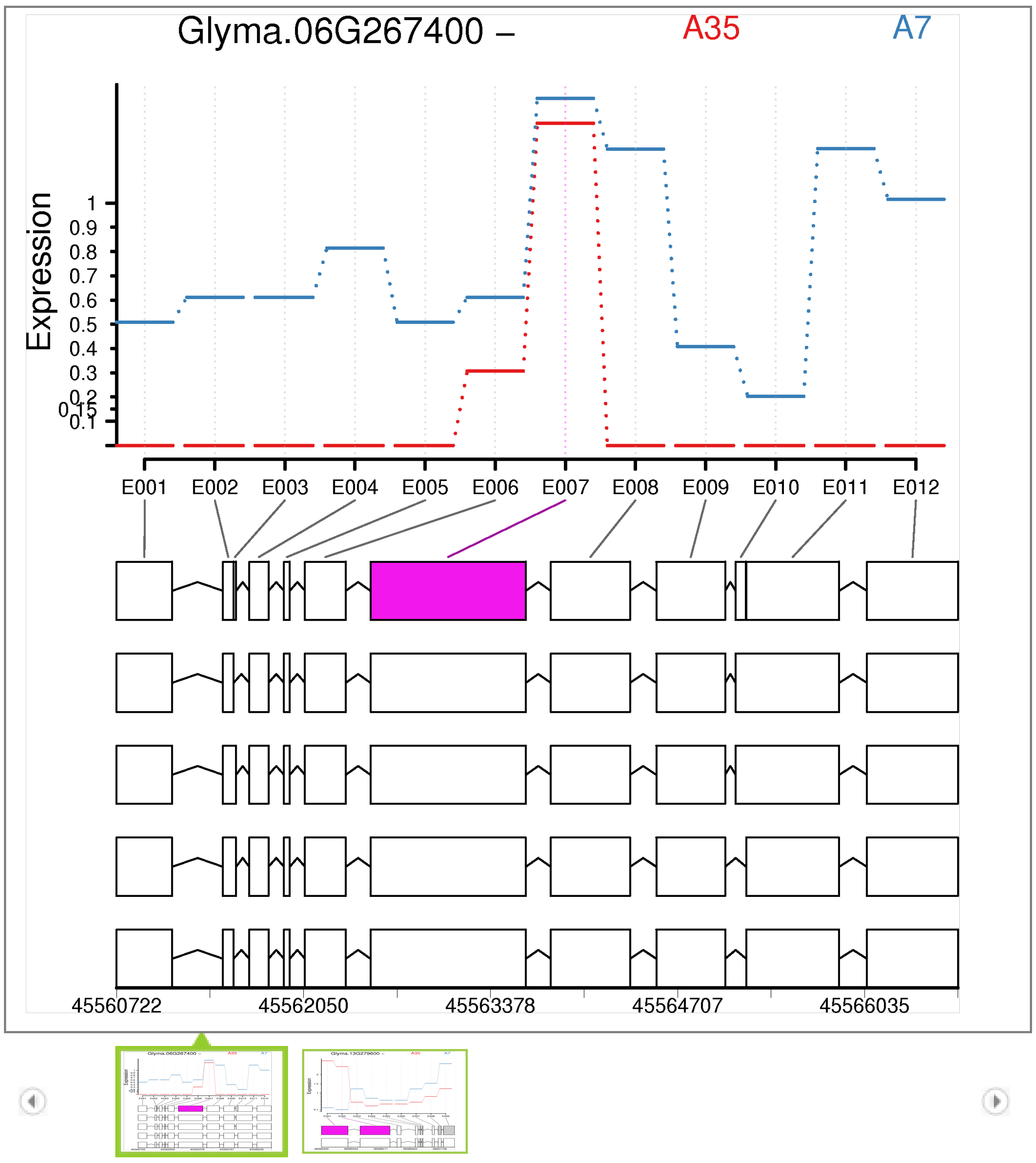

Figure 13.1 DEU基因外显子表达水平图，纵轴表示两组样本各自在外显子上的表达值，横轴表示外显子编号，差异外显子被紫红色高亮在这个外显子所在的转录本上面。其他的转录本是该转录本的可>变剪切。

四、参考文献

[1] Anders, S.(2010). HTSeq: Analysing high-throughput sequencing data with Python.(HTSeq)
[2] Anders, S., and Huber, W. (2010). Differential expression analysis for sequence count data. Genome Biol.(DESeq)
[3] Anders, S. and Huber, W. (2012). Differential expression of RNA-Seq data at the gene level-the DESeq package.(DESeq)
[4] Kim, D., G. Pertea, et al. (2012). TopHat2: Parallel mapping of transcriptomes to detect InDels, gene fusions, and more.(TopHat2)
[5] Langmead, B., Trapnell, C., Pop, M. & Salzberg, S.L. (2009). Ultrafast and memory-efficient alignment of short DNA sequences to the human genome. Genome Biol.(Bowtie)
[6] Langmead, B. and S. L. Salzberg (2012). Fast gapped-read alignment with Bowtie 2. Nature methods.(Bowtie 2)
[7] Marioni, J. C., C. E. Mason, et al. (2008). RNA-seq: an assessment of technical reproducibility and comparison with gene expression arrays. Genome research.
[8] Mortazavi, A., B. A. Williams, et al. (2008). Mapping and quantifying mammalian transcriptomes by RNA-Seq. Nature methods.
[9] Robinson, M. D., McCarthy, D. J. & Smyth, G. K. edgeR: a Bioconductor package for differential expression analysis of digital gene expression data. Bioinformatics.(edgeR)
[10] Trapnell, C. et al. (2010).Transcript assembly and quantification by RNA-seq reveals unannotated transcripts and isoform switching during cell differentiation. Nat. Biotechnol.(Cufflinks)
[11] Trapnell, C., Pachter, L., and Salzberg, S.L. (2009). TopHat: discovering splice junctions with RNA-Seq. Bioinformatics.(TopHat)
[12] Trapnell, C., A. Roberts, et al. (2012). Differential gene and transcript expression analysis of RNA-seq experiments with TopHat and

Cufflinks. nature protocols. (Tophat & Cufflinks)
[13] Wang, Z., M. Gerstein, et al. (2009). RNA-Seq: a revolutionary tool for transcriptomics. Nature Reviews Genetics.
[14] Love, M. I.,Anders, S., and Huber, W.(2015). Differential analysis of count data-the DESeq2 package. (DESeq2)
[15] Kim, D., Langmead, B., and Salzberg, S. L.,(2015). HISAT: a fast spliced aligner with low memory requirements. (HISAT)
[16] Pertea, M., Pertea, Geo. M., Antonescu, C. M., Chang, T. C., Mendell, J. T., and Salzberg, S. L.,(2015). StringTie enables improved reconstruction of a transcriptome from RNA-seq reads. (StringTie)

五、附录

1 文件目录列表

- Report
  - 00_Rawdata
  - 01_QC
  - 02_Mapping
  - 03_AS
  - 04_Novel
  - 05_SNV
  - 06_GeneExpression
  - 07_AdvancedQC
  - 08_DE
  - 09_GO
  - 10_KEGG
  - 11_DEU
  - 12_PCA

2 备注

结果文件建议使用Excel或者EditPlus等专业文本编辑器打开。

使用浏览器打开结题报告时，若出现类似"为了有利于保护安全性，Internet Explorer 已限制此网页运行可以访问计算机的脚本或ActiveX控件。请单击这里获取选项..."的提示，请选择允许。
